# Combination Immunotherapy as a Promising Strategy to Overcome Immunotherapy Resistance: From Emergence to Next‐Generation Approaches

**DOI:** 10.1002/advs.202518960

**Published:** 2026-02-25

**Authors:** Asmita Pandey, Sudhir Kumar Rai, Li Ma, Lauren Higa, Vedbar Khadka, Hua Yang, Youping Deng

**Affiliations:** ^1^ Department of Tropical Medicine, Medical Microbiology, and Pharmacology, John A. Burns School of Medicine University of Hawaii at Manoa Honolulu Hawaii USA; ^2^ Department of Quantitative Health Sciences John A. Burns School of Medicine University of Hawaii at Manoa Honolulu Hawaii USA; ^3^ Department of Molecular Biosciences and Bioengineering University of Hawaii at Manoa Honolulu Hawaii USA; ^4^ Genomics and Bioinformatics Shared Resource University of Hawaii Cancer Center Honolulu Hawaii USA; ^5^ Pacific Center for Genome Research University of Hawaiʻi at Manoa Honolulu Hawaii USA

**Keywords:** combination immunotherapy, immune checkpoint inhibitors, biomarkers, artificial intelligence, next generation immunotherapy

## Abstract

Advances in immunotherapy, particularly with immune checkpoint inhibitors, have improved clinical outcomes in various cancer subtypes; however, resistance often occurs because these treatments rely on single agents and show limited translational success when combined. Therefore, it is necessary to develop immune checkpoint inhibitor‐based combination strategies by integrating next‐generation immunotherapies and innovative biomarkers. This review highlights the emerging combinatorial approaches that can be tailored to the distinct tumor microenvironment types to overcome resistance to immune checkpoint inhibitors. It further emphasizes the rationale for integrating advanced biomarkers to predict response and personalize immunotherapy, highlighting the importance of combining next‐generation immunotherapies with immune checkpoint inhibitors and including them in current clinical trial designs. Building on this foundation, we integrate key lessons from failed clinical trials and the spectrum of toxicities associated with combination immunotherapy. Finally, we outline approaches for early biomarker discovery and clinical translation, including the incorporation of validated artificial intelligence–driven biomarker platforms into adaptive, biomarker‐guided trial designs.

## Graphical abstract

This review examines emerging combination immunotherapy strategies tailored to distinct tumor microenvironments and highlights next‐generation biomarkers that guide response prediction and treatment personalization. It integrates lessons from unsuccessful trials, addresses toxicity challenges, and outlines approaches for early biomarker discovery and adaptive trial design using advanced AI‐driven platforms.

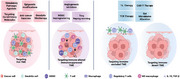

## 1 Introduction

**FIGURE 1:**
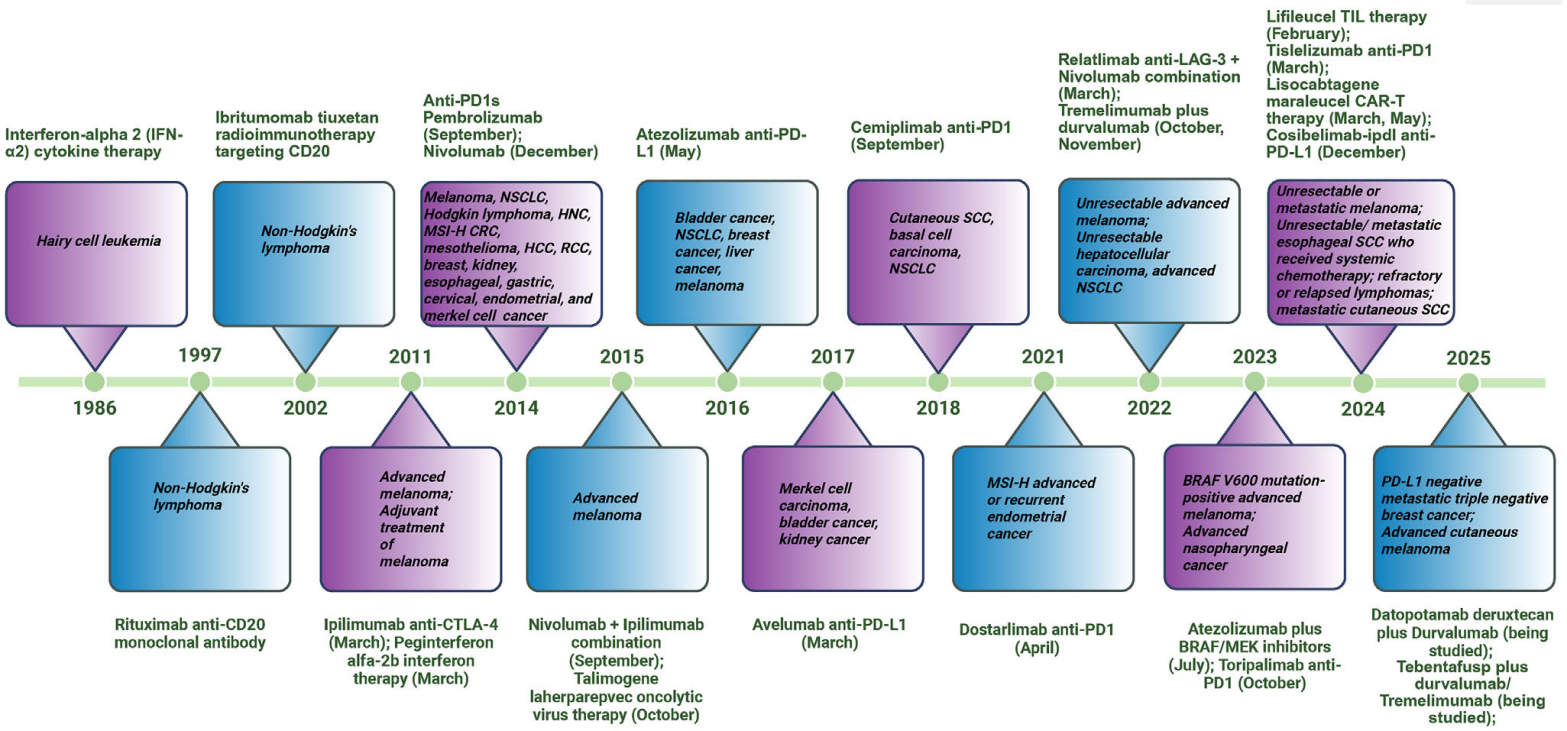
Major timeline of development of FDA‐approved immune checkpoint inhibitors and their combinations to date. HNC‐ head and neck cancer; CRC‐ colorectal cancer; HCC‐ hepatocellular carcinoma; RCC‐ renal cell carcinoma; SCC‐ squamous cell carcinoma. Created in BioRender. Mahmanzar, M. (2025) https://BioRender.com/3a642l2.

Immunotherapy has advanced cancer treatment by providing better clinical responses than traditional options such as surgery, chemotherapy, radiation, and targeted therapy [1]. The FDA's approval of single‐agent immune checkpoint inhibitors (ICIs) and combination therapies involving ICIs, including CAR‐T therapies, has revolutionized treatment. These therapies are continually being approved for additional indications and cancer stages. However, the approval rate for novel immunotherapeutic agents with breakthroughs has recently declined, despite promising results in animal studies and early trials [1, 2, 3, 4, 5, 6, 7, 8, 9, 10, 11, 12, 13, 14, 15, 16] (Figure 1). A major limitation of immunotherapies, especially ICIs, is treatment resistance in most patients, particularly in the monotherapy setting [1, 17]. Only a small fraction of patients initially respond to ICIs, with objective response rates of approximately 20%–30% in non–small cell lung cancer (NSCLC), 20%–45% in melanoma, and 20%–30% in renal cell carcinoma (RCC) [18, 19, 20, 21]. Moreover, over half of initial responders ultimately develop acquired resistance, underscoring the urgency for developing rational and multifaceted combination strategies [22, 23]. This highlights the need to explore novel, multifaceted combination strategies to address the dynamic nature of the tumor microenvironment (TME) [1, 17, 24]. Such strategies must be tailored to specific TME types, and the right pairing with immune checkpoint inhibitors (ICIs) can potentially turn a resistant TME into a responsive one.

Investigation and development of ICIs‐based combination immunotherapy also require a strong rationale and the inclusion of potential biomarker studies using high‐throughput methods [2, 25]. Leveraging high‐throughput technologies can facilitate novel hypothesis generation, strategic selection of combination agents by revealing mechanisms at cellular and molecular levels, and understanding heterogeneous treatment responses among patients by uncovering new predictive biomarkers [2, 25]. However, several biomarkers currently in use are not FDA‐approved, which necessitates clinical trials to validate their robustness and clinical potential [26]. Predictive biomarkers with high reliability can help optimize treatment strategies by identifying individuals who are more likely to respond to the therapy [1, 2]. Combining ICIs with other forms of immunotherapy, including next‐generation ICIs, while considering biomarkers, not only helps overcome resistance and identify patients most likely to benefit but also conserves time and resources.

In this review, we first summarize mechanisms of resistance to ICIs, anchored in contemporary frameworks of the different TME phenotypes, including immune‐inflamed, immune‐altered, immune‐excluded, and immune‐desert, and their translational implications. We then review rational combination strategies with ICIs to target different TME phenotypes to amplify T‐cell activation, remodel immunosuppressed TMEs, and convert non‐inflamed tumors to inflamed ones. Next, we integrate lessons from failed clinical trials and toxicities in combination immunotherapy. Finally, we describe approaches for early biomarker discovery and clinical deployment by integrating validated artificial intelligence (AI)‐based biomarker tools into adaptive, biomarker‐driven trial designs.

## 2 Resistance to Immune Checkpoint Inhibitors

Resistance to ICIs results from both tumor‐intrinsic factors and elements of the tumor microenvironment, including TME architecture and cellular heterogeneity [27]. There are two main types of resistance: i) primary resistance and ii) acquired resistance. Primary resistance occurs when the clinical population does not initially respond to ICIs [17]. Within the TME, chronic antigenic stimulation leads to T cell exhaustion. Although T cell exhaustion can sometimes predict response to ICIs, severe exhaustion and the upregulation of exhaustion and co‐inhibitory checkpoint molecules can cause resistance [28]. Exhausted T cells have distinct epigenetic programs that hinder ICIs from rejuvenating T cells, even when they express PD1 [29]. Regulatory T cells (Tregs) infiltrated within the TME create an immunosuppressive environment that is further reinforced by the infiltration of other suppressive cells, such as MDSCs, VEGF, cancer‐associated fibroblasts (CAFs), tumor‐associated macrophages (TAMs), neutrophils, and dendritic cells [30]. Consequently, the TME, as a complex environment composed of various cellular networks, impedes the development of cell‐mediated immunity at multiple stages, thereby reducing the effectiveness of immunotherapy.

**FIGURE 2:**
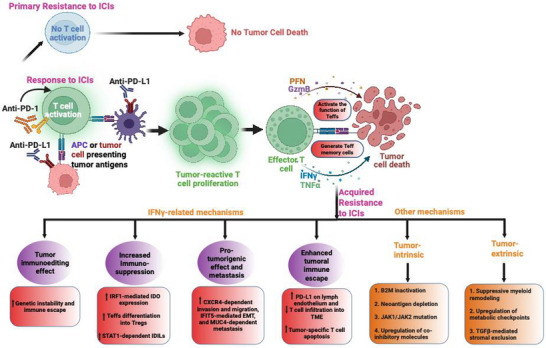
Mechanism of action of ICIs in response and in IFNγ‐driven resistance. In response, cytotoxic T lymphocytes (CTLs) are primed and activated, followed by effector T cells killing of tumor cells in the presence of IFNγ, TNFα, granzyme B, and perforin, which generates effector memory T cells. Multiple tumor intrinsic and extrinsic mechanisms cause acquired resistance to ICIs, and one of the leading mechanisms involve chronically exposed or CpG‐hypermethylated IFNγ that causes resistance to ICIs by several mechanisms: i) tumor immune escape by upregulating PD‐L1, reducing T cell infiltration, and increasing tumor‐specific T cells apoptosis; ii) CXCR4‐mediated increased migration, IFIT5‐mediated epithelial‐mesenchymal transition (EMT), and mucin4 (MUC4)‐dependent metastasis; iii) enhanced immunosuppression by increasing IRF1‐mediated indoleamine 2,3‐dioxygenase (IDO) expression, differentiation of effector T cells into regulatory T cells, and increased STAT1‐dependent interferon‐derived inhibitory ligands (IDILs); and iv) increased genetic instability resulting in tumor immune escape. Created in BioRender. Mahmanzar, M. (2025) https://BioRender.com/cordqwu.

Acquired resistance refers to a clinical population that initially responds to ICIs but later develops resistance, leading to disease progression [18]. While IFNγ is the main factor promoting ICIs response, antigen presentation, and PD‐L1 expression, defects in IFNγ production or mutations/downregulation of JAK1/2 can allow tumors to escape IFNγ signaling, resulting in cancer cell proliferation [17, 30](Figure 2). Recent studies show that chronic low levels of IFNγ promote metastasis by increasing ICAM‐1 and CD133, while higher concentrations induce anti‐tumor effects, suggesting that IFNγ levels influence the tumor microenvironment (TME) [31]. In the TME, IFNγ has dual roles: it can promote tumor destruction by acting as a cytotoxic cytokine with granzyme B and perforin, but it can also support immune suppression by stimulating production of checkpoint molecules like PD‐L1, IDO, PD–L2, CTLA4, and Foxp3 [31], which reduce the tumor‐killing capacity of T cells and NK cells [32]. Additionally, IFNγ can promote metastasis through CXCR4‐mediated pathways and induce epithelial‐mesenchymal transition (EMT) via JAK/STAT1 and IFIT5 [33]. It can also increase metastasis by decreasing tumor suppressors like Elf5 and FBXW7 [31]. One reported mechanism of IFNγ‐related resistance to ICIs involves increased STAT1‐induced inhibitory ligands, which promote immune evasion [31]. Upregulation of immune checkpoint molecules can lead to T cell apoptosis, impairing the anti‐tumor immune response and fostering resistance to ICIs [30]. Another mechanism is that IFNγ can cause genetic instability in tumors, promoting immune escape. It has been shown that IFNγ‐treated tumor cells can induce immunosuppression via TNF‐ and NF‐kB pathways by increasing receptor‐interacting protein kinase 1 (RIPK1) [34]. In lymph nodes, chronic IFNγ exposure can drive regulatory T cell (Treg) differentiation and facilitate metastasis [35]. Recent evidence indicates that IFNγ enhances YAP‐driven adaptive resistance to anti‐PD1 therapy [36]. Inhibiting tumor IFNγ signaling has been reported to improve ICIs response by increasing cytotoxic T cell infiltration and boosting anti‐tumor activity from T cells and NK cells, especially in tumors that were previously resistant. In addition to IFNγ‐related mechanisms, acquired resistance to ICIs is recognized as a multifactorial process driven by both tumor‐intrinsic and microenvironmental adaptations. Tumor‐intrinsic alterations, including loss of antigen presentation through B2M inactivation, neoantigen depletion, and JAK1/JAK2 mutations, have been repeatedly identified in progressing lesions and diminish tumor visibility to cytotoxic lymphocytes [37]. Upregulation of alternative inhibitory pathways such as TIM3, LAG3, and TIGIT contributes to T‐cell re‐exhaustion and functional impairment despite PD1/PD‐L1 blockade [38]. Tumor‐extrinsic mechanisms, such as suppressive myeloid remodeling (MDSCs, M2‐like TAMs), metabolic checkpoints such as IDO1, and TGF‐β–driven stromal exclusion further support an immune‐refractory microenvironment that limits durable responses [39]. Longitudinal genomic and transcriptomic analyses also demonstrate neoantigen loss, EMT, and non‐genetic plasticity as additional contributors to relapse following initial response [40]. Collectively, these findings highlight multifactorial axes of resistance, and effective therapeutic strategies require considering strategies involving immune cell activation and exhaustion in TME, antigen‐presentation status, alternative checkpoint pathways, and suppressive TME states in a rational combination design.

## 3 TME Phenotypes

**FIGURE 3:**
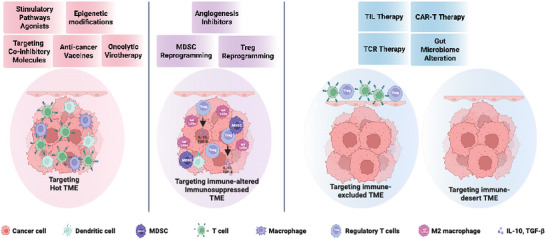
Combination strategies with ICIs to target different TMEs. Hot TME is targeted with ICIs in combination with co‐stimulatory agonists, epigenetic alteration, blocking co‐inhibitory molecules, cancer vaccines, and oncolytic viruses. Immunosuppressed TME is targeted with ICIs, combining with angiogenesis inhibition, and MDSCs/Treg reprogramming. Cold TMEs, such as immune‐excluded and immune‐desert TME is targeted by ICIs in combination with microbiome and adoptive therapies, including TILs, CAR‐T, and TCR therapies. Created in BioRender. Mahmanzar, M. (2025) https://BioRender.com/82kt77n.

In TME, solid tumors are classified into three distinct types based on spatial distribution and density of lymphocyte infiltration within the tumor and its stromal margin: 1) immune‐inflamed hot tumors, 2) immune‐altered immunosuppressive tumors, and 3) cold tumors, such as immune‐excluded tumors and immune‐desert tumors [41]. Hot tumors often contain significant numbers of infiltrating effector immune cells, pro‐inflammatory cytokines, and tumor mutational burden, which make them mostly responsive to immunotherapy. Although the level of tumor‐infiltrating lymphocytes (TILs) does not always predict the response to immunotherapy, hot TME can better facilitate this response compared to less immunogenic TME [1]. Immunosuppressive tumors have substantial immune infiltration, yet T cells are dysfunctional due to dominant inhibitory molecules within the TME. These include Tregs, MDSCs, TAMs, and cytokines such as IL‐10 or TGF‐β [40, 41]. The immune‐excluded cold tumor phenotype occupies a biologically and clinically distinct space, for example, immune cells are present but are confined to the stroma or the peritumoral margin and fail to penetrate the tumor parenchyma, often due to physical or vascular barriers, stromal architecture, or suppressive signals [40, 41, 42]. In contrast, immune‐desert cold tumors are characterized by a paucity or absence of lymphocytes both in the tumor core and at the invasive margin, consistent with a lack of pre‐existing antitumor immunity [43, 44]. Immunotherapeutic treatment of these distinct tumor phenotypes requires specific immunotherapy combinations to achieve better clinical outcomes (Figure 3, Table 1).

**TABLE 1:** Combination Therapeutic Strategies to Enhance Immunotherapy Efficacy in Various TMEs.

TME Target for Combination Immunotherapy	Innovative Combination Strategies with existing ICIs	Proposed Mechanisms of Action	Synergistic Potential with Established ICIs	Clinical Development Status	Representative Indications
**Immune‐inflamed hot TME**	Next‐generation ICIs [24, 45, 55, 62, 64, 65]	Inhibition of multiple inhibitory pathways by alternative immune checkpoints to reinvigorate T cell function and persistence	Synergistic effects with anti‐PD‐1/PD‐L1 and anti‐CTLA‐4 therapies, potentially overcoming resistance	FDA‐approved (relatlimab + nivolumab) + phase I‐III trials	FDA‐approved (melanoma) + phase I‐III trials (NSCLC, SCLC, multiple solid and hematological cancers)
	Stimulatory signals activators [68, 73, 82, 84, 92]	Promotes activation of innate and adaptive immune responses to enhance tumor‐killing	Complements PD‐1/PD‐L1 blockade by boosting anti‐tumor immunity	Phase I‐III trials	Phase I‐III trials (Advanced solid tumors, pancreatic cancer, melanoma)
	Anti‐cancer vaccines [97, 99, 105]	Enhances antigen presentation and primes tumor‐specific T cells	Synergizes with ICIs by increasing the pool of tumor‐reactive T cells	Phase I‐III trials	Phase I‐III trials (Melanoma, glioblastoma, pancreatic cancer, prostate cancer)
	Oncolytic virotherapy [107, 109]	Induction of immunogenic cell death and enhancement of antigen presentation	Enhances antigen release and inflammatory response to increase ICIs sensitivity	FDA‐approved (T‐VEC) + phase I‐III trials	FDA‐approved (melanoma) + phase I‐III trials (melanoma, glioma)
	Epigenetic modification [114, 115]	Upregulation of tumor antigen expression and enhancement of T cell infiltration	Can sensitize tumors to ICIs therapy by altering the immunogenicity of cancer cells	Phase I‐II trials	Phase I‐II trials (Colorectal cancer, ovarian cancer, other solid tumors)
**Immune‐altered immunosuppressed TME**	MDSCs reprogramming [122, 123, 124]	Reduces immunosuppressive myeloid cell activity in TME	Enhances T cell activation and efficacy of PD‐1/PD‐L1 blockade	Phase I‐II trials	Phase I‐II trials (Pancreatic cancer, prostate cancer)
	Tregs reprogramming [130]	Depletes or reprograms immunosuppressive regulatory T cells	May improve responses to ICIs by reducing Treg‐mediated suppression of effector T cells	Phase I‐II trials	Phase I‐II trials (Ovarian cancer, hepatobiliary cancer)
	Angiogenesis inhibitors [132, 133, 135]	Normalizes tumor vasculature to improve immune cell infiltration	Can improve the delivery and efficacy of ICIs by modulating the TME	FDA‐approved (Bevacizumab + atezolizumab, Pembrolizumab + axitinib, pembrolizumab + lenvatinib) + phase II‐III trials	FDA‐approved (NSCLC, HCC, renal cell carcinoma) + phase II‐III trials (multiple solid tumors)
**Immune‐excluded and immune‐desert cold TME**	TIL therapy [16, 140, 143]	Provides ex vivo‐expanded tumor‐reactive T cells	Potential to convert "cold" tumors to "hot," enhancing ICIs responsiveness	FDA‐approved (Lifileucel) + phase I‐III trials	FDA‐approved (melanoma) + phase I‐III trials (cervical cancer, lung cancer, HNSCC, gastrointestinal cancer)
	CAR‐T therapy [148, 149]	Introduction of engineered T cells with tumor‐specific receptors	Complements ICIs by providing antigen‐specific immune activation	FDA‐approved (lisocabtagene maraleucel, tisagenlecleucel, axicabtagene ciloleucel, brexucabtagene autoleucel, idecabtagene vicleucel, ciltacabtagene autoleucel, obecabtagene autoleucel) + phase I‐II trials	FDA‐approved (B cell malignancies) + phase I‐II trials (solid tumors such as glioblastoma, sarcoma and pancreatic cancer)
	TCR therapy [155, 232]	Infusion of T cells with engineered TCRs specific for tumor antigens	Can complement ICIs therapy by introducing tumor‐reactive T cells into the TME	FDA‐approved (Tebentafusp) + phase I‐II trials	FDA‐approved (HLA‐A*02:01‐positive unresectable/metastatic uveal melanoma) + phase I‐II trials (cutaneous melanoma)
	Gut flora alteration [214, 215]	Modulates gut microbiota to improve systemic anti‐tumor response	Enhances response to ICIs by promoting immune‐favorable microbiome composition	Phase I‐II trials	Phase I‐II trials (melanoma, NSCLC, colorectal cancer, gastrointestinal malignancies)
**Non‐Immunogenic TME to Enhance the Production of Tumor‐Specific Antigens**	Chemotherapy [1, 161, 163, 164]	Induction of immunogenic cell death and release of tumor antigens for immune activation	Synergizes with ICIs by boosting tumor immunogenicity	FDA‐approved (Pembrolizumab + platinum‐based chemotherapies) + phase II‐III trials	FDA‐approved (NSCLC) + phase II‐III trials (lung cancer, TNBC, gastroesophageal cancer, bladder cancer)
	Radiotherapy [168, 169, 171, 172]	Enhancement of tumor antigen presentation and T cell priming	Potential to improve ICIs efficacy through abscopal effects and increased tumor immunogenicity	Phase I‐III trials	Phase I‐III trials (NSCLC, melanoma, prostate cancer)
	Targeted therapy [175, 176, 179, 180]	Modulation of oncogenic pathways to alter tumor immunogenicity	May enhance ICIs responses by changing the tumor's immune contexture	FDA‐approved (Tyrosine kinase inhibitors + anti‐PD1/PD‐L1, HER2‐targeted antibody + anti‐PD1) + phase I‐III (BRAF/MEK inhibitors + anti‐PD1)	FDA‐approved (RCC, endometrial cancer, HER2+ gastroesophageal cancer) + phase I‐III trials (NSCLC, cholangiocarcinoma)
	Small molecule‐based targeted therapy [182, 184, 185]	Inhibition of specific molecular pathways to enhance anti‐tumor immunity	Can complement ICIs therapy by targeting non‐redundant immunosuppressive mechanisms	Phase I‐III trials	Phase I‐III trials (melanoma, multiple solid tumors)

## 4 Targeting Hot TME for T Cell Activation

### 4.1 State‐of‐the‐Art Combination Strategies With Next‐Generation ICIs

***Anti‐LAG3***: Besides activated lymphocytes and plasmacytoid dendritic cells (DCs), lymphocyte activation gene‐3 (LAG3) is highly expressed on exhausted T cells. LAG3, which is found on the surface of Tregs, can suppress DC activity by binding to MHC‐II [2]. Relatlimab, the first FDA‐approved LAG3 inhibitor for treating metastatic melanoma in combination with nivolumab for patients over 12 years old, reduces regulatory T cell‐mediated immunosuppressive effects by binding to its ligand and enhances the expansion and killing activity of effector T cells [46]. The clinical success of LAG3 inhibitors has increased interest in LAG3‐based ICIs for treating other solid cancers, including relatlimab and eftilagimod alpha in phase II trials, especially for non‐small cell lung cancer (NSCLC) [47]. Moreover, combining anti‐LAG3 with other ICIs like anti‐PD1 has shown synergistic effects in preclinical mouse models [48]. Although nivolumab‐relatlimab has shown limited clinical benefit in anti‐PD1‐treated melanoma, this combination as first‐line therapy in a phase II/III trial in RELATIVITY‐047 resulted in longer progression‐free survival (PFS) for patients, regardless of PD‐L1/LAG3 expression levels, compared to nivolumab alone in advanced melanoma [49]. The nivolumab‐relatlimab combination is also being studied as adjuvant and neoadjuvant therapy [49, 50]. It is being tested alongside two other immunotherapies, sarilumab and ipilimumab, for multiple solid and hematological cancers, including advanced and metastatic melanoma [52]. Combining inhibitors targeting co‐inhibitory molecules such as LAG3 with immune checkpoint inhibitors aims to target hot TME (Figure 3).

***Anti‐TIGIT***: TIGIT (T‐cell immunoreceptor with Ig and ITIM domains) is a co‐inhibitory receptor expressed on the surface of activated, memory, regulatory, follicular helper, and NK T cells, including NK cells [52, 53]. TIGIT, upon binding to its ligand CD155, expressed on tumor and antigen‐presenting cells (APCs), halts immune activation mediated by CD226 [54]. Ongoing anti‐TIGIT‐based clinical trials of tiragolumab have shown promising results alongside existing ICIs, such as atezolizumab, in NSCLC patients with PD‐L1>1% [56]. In a phase‐II trial of CITYSCAPE, PD‐L1‐selected NSCLC patients treated with first‐line tiragolumab plus atezolizumab demonstrated better ORR and longer PFS compared to placebo and atezolizumab alone [56]. Similarly, the neoadjuvant trial of KEYMAKER‐U02 revealed that vibostolimab combined with pembrolizumab improved relapse‐free survival compared to pembrolizumab monotherapy [57]. Anti‐TIGIT clinical trials have also encountered setbacks in late‐stage development. For example, the Phase III SKYSCRAPER‐01 and ‐02 studies evaluating tiragolumab in combination with atezolizumab failed to meet their primary endpoints, including in patients with PD‐L1–high non–small cell lung cancer [58]. Furthermore, adding chemotherapy as the initial treatment in the same combination did not improve outcomes in PD‐L1‐high advanced squamous cell lung cancer (SCLC) [59]. These failures can be explained by several reasons, such as the absence of prospective biomarker enrichment, since heterogeneous TIGIT/PD‐L1 co‐expression in broad Phase‐III populations can dilute true responders, and overreliance on small Phase‐II signals without supporting pharmacodynamic or mechanistic biomarkers. This illustrates systematic challenges in advancing combination immunotherapies and highlights the need for prospective biomarker selection and mechanistic concordance before Phase‐III escalation. TIGIT may also serve as a potential biomarker for predicting clinical response, as higher Tregs‐derived TIGIT to CD226 ratios predicted poorer outcomes in patients with advanced melanoma treated with ICIs [59], warranting further attention.

***Anti‐TIM3***: TIM3 (T‐cell immunoglobulin and mucin‐domain containing molecule 3), a receptor mainly expressed on CD4+/CD8+/regulatory T cells and other immune cells such as DCs, NK cells, monocytes, and macrophages, has multiple functions. It binds to various ligands, including galectin‐9, which leads to Th1 cell apoptosis, and carcinoembryonic antigen‐related cell adhesion molecule 1 (CEACAM1), which is involved in regulating anti‐tumor immunity. Additionally, phosphatidylserine promotes immune tolerance by clearing apoptotic bodies [2, 24, 60]. Besides its co‐inhibitory role, TIM3 also participates in co‐stimulating T cells through interaction with BAT3, a transcription factor associated with HLA‐B [25]. Although higher TIM3 expression has been linked to poorer overall survival, its value as a prognostic biomarker requires further investigation [62]. In a phase‐I trial, combining sabatolimab with anti‐PD1 spartalizumab showed an enhanced anti‐tumor response, leading to ongoing phase‐II studies [63]. Similarly, cobolimab, another anti‐TIM3 antibody, was reported to boost anti‐cancer immunity when combined with anti‐PD1 dostarlimab, and this combination is currently in a phase‐II trial for hepatocarcinoma [64]. While anti‐TIM3 treatment activates IFN‐γ‐producing CD8+ T cells, multiple clinical trials are exploring combinations of anti‐TIM3 with CTLA4 or PD1 inhibitors [61]. Targeting TIM3 alongside existing ICIs could be beneficial in overcoming resistance to current therapies.

***Anti‐TIM4***: T‐cell immunoglobulin and mucin‐domain containing molecule 4 (TIM4) is another co‐inhibitory immune checkpoint receptor for phosphatidylserine, highly expressed on the surface of cavity‐resident macrophages, and functions as a suppressor of CD8+ T cell anti‐tumor activity [65]. In pleura‐ and peritoneum cavity‐derived samples, patients with advanced NSCLC who received ICIs showed that TIM4 upregulation was correlated with CD8+ T cells sequestration distant from the tumor site, thereby hindering their proliferation [65]. Anti‐Tim‐4 treatment reversed the CD8+ T cell sequestration and enhanced anti‐tumor response while facilitating the expansion of CD8+ T cells in mouse models [65]. Continued investigation of TIM‐4–targeted strategies may offer a promising avenue for augmenting clinical responses when combining with existing immune checkpoint inhibitors.

***Other next‐generation ICIs***: VISTA (V‐domain immunoglobulin suppressor of T‐cell activation), a receptor for ligands VSIG‐3 and PSGL‐1, can inhibit effector T cell function through its interaction with its ligand; therefore, blocking VISTA activates T cells and leads to the production of pro‐inflammatory cytokines [65, 66]. VISTA is also reported to promote immunosuppression by supporting Treg cell survival and impairing APC function [67]. Further study of this immune checkpoint molecule could provide insight into its broad role in anti‐tumor immunity, supporting its clinical development. Additionally, other inhibitory immune checkpoint molecules are being explored as potential targets, including SIRPα (signal regulatory protein α), which interacts with CD47; BTLA (B and T lymphocyte attenuator), which binds to HVEM; LILRB4 (leukocyte immunoglobulin‐like receptor subfamily B member 4), which interacts with ApoE and other ligands; and Siglec‐7 (sialic acid‐binding immunoglobulin‐like lectin 7), which binds to sialic acid‐containing glycan ligands [25].

### 4.2 Combination Strategies With Stimulatory Signal Activators

***Stimulatory pathway activation***: Activating stimulatory pathways by targeting co‐stimulatory molecules, such as Toll‐like receptors (TLRs), inducible T‐cell costimulator (ICOS), CD40, and OX40, can be an effective strategy to overcome inhibitory signals [1, 2]. ICOS, a member of the CD28 superfamily, aids in activating effector T cells, interacting with B cells, and infiltrating Tregs [68]. An ICOS‐agonist combined with anti‐CTLA4 improved the outcomes of anti‐CTLA4 treatment in a preclinical model [69]. Other ICOS‐agonists paired with anti‐PD1 are under clinical trials for solid tumor treatment [1]. OX40, part of the TNF receptor superfamily and transiently expressed on antigen‐stimulated CD4+/CD8+ T cells, plays a role in activating and proliferating effector T cells while promoting their survival and tumor‐killing activity, including Treg development [2]. By inhibiting Treg activity, OX40 agonists are reported to modestly boost immunogenicity as standalone agents and may have greater potential when combined with strategies that increase immune repertoire and enhance immunogenicity within the tumor microenvironment, due to their temporary expression [70]. Other co‐stimulatory molecules such as CD27/28, 41BB, and glucocorticoid‐induced TNFR‐related protein (GITR) are currently under investigation [71]. Proper optimization of sequencing and combination choices for these agents could significantly improve current clinical outcomes [71].

***Innate immunity agonists***: Enhancing activating signals to boost innate immunity by using agonists for signaling receptors, such as TLRs, RLRs, and STING, has been a key research focus. Antigen‐presenting cells detect and respond to stimuli like DAMPs and PAMPs through signaling receptors such as TLRs and RLRs [2]. Currently, multiple TLR agonists and antagonists are in clinical trials, including anti‐PD1 agents like CMP‐001, a TLR‐9 agonist used for various tumors, including lymphoma and melanoma [71, 72]. This combination has shown promising results in patients with metastatic and refractory melanoma [74] and has produced a favorable clinical response as neoadjuvant therapy in resectable melanoma [75]. SD‐101, another TLR‐9 agonist, is being tested with anti‐PD1 in advanced melanoma and with radiation plus targeted therapy in lymphoma [75, 76]. TLR‐9, which is mainly expressed by APCs, detects and responds to dsDNA, leading to repolarization of M2 macrophages toward M1 phenotype and the production of TNF‐ and IL‐12, resulting in a pro‐inflammatory TME. Therefore, using TLR‐9 agonists enhances these responses to strengthen anti‐tumor immunity [2]. TLR‐7 agonists are combined with anti‐PD1 for melanoma and with chemotherapy plus radiation for breast cancer [77, 78]. The phase I/II trial of a TLR‐7/8 agonist with pembrolizumab for advanced solid cancers is currently ongoing [78, 79].

STING (Stimulator of Interferon Genes) is essential for inducing type‐I IFNs, which play a key role as a pro‐inflammatory step in generating effective anti‐tumor immunity [81]. STING agonists can boost DC‐mediated antigen presentation, T cell activation, and NK cell‐mediated tumor cell killing by decreasing MDSC‐driven immunosuppression [2]. Several clinical trials, involving STING activators either alone or in combination, are currently underway for advanced cancers [82]. Intratumoral delivery of the STING agonist SYNB1891 in NCT04167137 demonstrated a tolerable safety profile in treating advanced human cancers, both as a standalone therapy and combined with atezolizumab [83].

***Cytokine enhancement***: Advances in biomedical engineering have led to the development of novel cytokine delivery strategies, such as PEG‐based IL‐2 (NKTR‐214), which demonstrated safer outcomes with low toxicity and increased effector immune cell infiltration compared to IL‐2 alone [84]. Combination strategies of NKTR‐214 with anti‐PD1/CTLA4, nemvaleukin‐α, STK‐012, GM‐CSF, and IL‐12 are under study, and some have shown good safety profiles [70, 71, 72, 73, 74, 75, 76, 77, 78, 79, 80, 81, 82, 83, 84, 85, 86, 87, 88, 89, 90]. In TME, the anti‐tumor activity is significantly diminished by immunosuppressive TGFβ, which has been targeted using various combination approaches, including immunotherapy [2]. One promising method to target TGFβ is to block the p38 pathway, such as with small molecule inhibitors combined with ICIs, which have shown enhanced dendritic cell (DC) maturation and reduced immunosuppressive cytokine levels [90]. Additionally, inhibiting receptors, such as CXCR2 or CXCR4, can reduce immunosuppression in the TME by lowering Treg levels, while engineered cytokines such as CCL3 and CXCL11 can boost effector cell infiltration [91]. Both chemokine receptor inhibitors and engineered chemokines, whether alone or combined with ICIs, virotherapies, and cellular therapies, are currently under investigation [2, 91].

***Bispecific antibodies***: Bispecific antibodies that bind simultaneously to T cells and other immune effector cells have been a major area of focus. Tebentafusp, a T cell receptor bispecific antibody that interacts with CD3 and gp‐100, has been approved for treating HLA‐A*02:01‐positive metastatic melanoma [93]. The Phase III trial showed a 6‐month overall survival (OS) benefit in patients treated with tebentafusp, regardless of gp‐100 expression, and this survival benefit persisted long term after a 3‐year analysis [93]. The study combining tebentafusp with durvalumab demonstrated a consistent safety profile, similar to that of monotherapies, at the highest target doses, while also showing clinical efficacy in anti‐PD‐L1‐resistant metastatic cutaneous melanoma. Several other combinations of ImmTACs class agents with ICIs are currently in clinical trials, such as IMC‐C103C with atezolizumab and brenetafusp/IMC‐F106C with ICIs to treat advanced solid cancers with HLA‐A2 tissue expression [93, 94]. Recently, blinatumomab, a bispecific antibody that binds to CD19 on tumor cells and CD3 on T cells, showed relatively longer overall survival and remission rates in patients with CD19‐positive acute B‐cell lymphoblastic leukemia, earning FDA approval [96]. The combination of blinatumomab with ICIs, like pembrolizumab, is currently under investigation [97].

### 4.3 Combination Strategies With Anti‐Cancer Vaccines

***Dendritic cell‐based vaccines***: Cancer vaccines consist of cells, proteins or peptides, oncolytic viruses, and nucleic acids [1]. The first FDA‐approved dendritic cell‐based vaccine, sipuleucel‐T, along with other cell‐based vaccines like oncovax and GVAX, which are currently under investigation, use individual patient's inactivated tumor cells as antigens [1]. These vaccines, especially when combined with ICIs, such as anti‐CTLA‐4 and anti‐PD1/PD‐L1, have shown promising clinical benefits, supporting further development of this combination strategy [98].

***Peptide and nucleic acid‐based vaccines***: Vaccines using nucleic acids, such as DNA or RNA, are taken up by antigen‐presenting cells and converted into tumor‐specific antigens [1]. Delivering these nucleic acids effectively remains challenging, often resulting in less translation into antigen proteins and lower antigen presentation [1]. Recently, new mRNA vaccine strategies have shown a better safety and immunogenicity profile [99]. The main focus of current mRNA vaccine efforts is on optimizing formulation and sequencing with immunotherapy, where vaccination prior to immunotherapy has led to improved clinical outcomes [100]. In a phase‐II clinical trial, personalized mRNA vaccination before anti‐PD1 therapy improved relapse‐free survival in melanoma patients, demonstrating durable neoantigen‐specific CD8^+^ T‐cell expansion and long‐term clinical benefit [100, 101]. Additional early‐phase studies summarized in recent reviews indicate feasibility and immunogenicity of personalized mRNA vaccines in traditionally less immunogenic indications such as pancreatic cancer and glioblastoma, although efficacy signals remain preliminary [102, 103]. Remaining challenges include robust prediction of antigen presentation and neoantigen immunogenicity, optimal sequencing and dosing with ICIs, and scalable manufacturing. Continued refinement of formulation chemistry, standardized immunomonitoring, and integration with biomarker‐driven combination trials will be essential to translate mRNA vaccine platforms across indications.

***Personalized neoantigen vaccines***: Since neoantigens are unique to tumor cells, they can be identified through sequencing by comparing tumor cells with PBMCs using high‐throughput methods [1]. The target antigens, encoded by cells, nucleic acids, or peptides, can be combined with adjuvants to enhance the immune response. Once delivered and taken up by antigen‐presenting cells (APCs), these cells transport neoantigens to the lymph nodes and present them to naive T cells. This process prompts naive T cells to differentiate into effector T cells that are specific to neoantigens, which then infiltrate the tumor via blood vessels [1]. Neoantigen vaccines based on dendritic cells with higher affinity for *HLA‐A‐02:01* demonstrate an increased diversity in the T cell receptor repertoire that is specific to neoantigens [105]. A study involving five melanoma patients reports that an RNA‐based neoantigen vaccine elicited a strong neoantigen‐specific T cell response [106], suggesting that personalized neoantigen vaccines could become a powerful part of future cancer immunotherapy. It is also crucial to standardize neoantigen prediction methods to advance vaccine platforms and improve understanding of immune cell interactions in vaccinated environments.

### 4.4 Combination Strategies With Oncolytic Virotherapy

Oncolytic viruses disrupt tumor blood vessels, and since cancer patients often have poorly functional blood vessels, improving vasculature with certain drugs can boost the response to oncolytic viruses [107]. The first oncolytic virus, T‐VEC, combined with Pembrolizumab, increased the objective response rate of Pembrolizumab, with responses linked to TIL infiltration and IFNγ expression after treatment [108]. However, this combination showed limited survival benefits in a phase‐III trial [109]. Clinical trials with another oncolytic virus, ONCOS‐102, an engineered adenovirus expressing GM‐CSF, combined with cyclophosphamide and other anti‐cancer drugs are ongoing for various cancer types [1]. Different administration methods, such as intratumoral and systemic, and various combination strategies, including those with anti‐PD1 to boost the innate immune response, are under investigation [2]. RP1, an HSV‐1‐based oncovirus, showed improved ORR and CR in melanoma patients resistant to anti‐PD1 when combined with nivolumab [110]. Other oncolytic viruses, including anti‐CTLA4‐expressing RP2 and CD40L‐ and 41BBL‐expressing RP3, are being studied, either alone or with anti‐PD1 [110, 111]. Additionally, a Phase‐I trial is underway for T3011, an HSV‐1‐based oncolytic virus expressing IL‐12 and anti‐PD1(Fab), tested alone or with anti‐PD1 to treat advanced solid cancers [112].

### 4.5 Combination Strategies With Epigenetic Modification

Epigenetic modifications in tumor cells can suppress tumor antigen expression, allowing the cancer to evade immune detection. For instance, DNA methylation can decrease NY‐ESO‐1 expression, while histone acetylation increases it [113]. The demethylation approach has been shown to boost NY‐ESO‐1 expression and enhance CD8+ T cell responses [114]. Preclinical models of ovarian cancer demonstrated that epigenetic changes in chemokines lead to altered Th1 chemokine‐mediated T cell trafficking [115]. Combining epigenetic modifiers, such as the demethylating agent 5‐aza‐2′‐deoxycytidine, with various immunotherapies, including ICIs, is being tested to improve responses across multiple cancers, including NSCLC [116]. Epigenetic modifications may promote tumor antigen expression and enhance T cell activity, such as rejuvenating exhausted T cells [117]. Studies in preclinical models suggest that histone deacetylase inhibitors combined with ICIs could boost anti‐tumor responses by preventing CD4+ T cell apoptosis [118].

Overall, the most clinically mature strategies for inflamed (hot) tumors are checkpoint combinations with proven Phase‐III evidence, such as oncolytic (T‐VEC) or vaccine approaches that can demonstrably increase intratumoral T cell infiltration [118, 119]. Next‐generation co‐stimulatory agonists and novel checkpoint antibodies remain promising but should advance only with validated pharmacodynamic markers and early safety/sequence optimization.

## 5 Targeting Immunosuppressed TME

Immune tolerance helps maintain immune homeostasis by stopping immune cell activation, which is crucial to prevent unnecessary inflammation in the host. However, this mechanism, mediated not only by ICIs but also by immunosuppressive molecules such as MDSCs, including TAMs, regulatory T cells, and abnormal APCs, promotes the suppression of anti‐tumor immunity [121].

### 5.1 Combination Strategies With Reprogrammed MDSCs

T cell inhibitory cytokines and reactive oxygen/nitrogen species are produced during myeloid‐derived suppressor cells (MDSCs) development, thereby suppressing anti‐tumor immunity, promoting metastasis, and creating resistance to anti‐cancer treatments, including immunotherapy [1]. MDSC infiltration in TME is positively associated with survival as well as with poor prognosis [121]. The increased presence of tumor‐associated neutrophils (TANs) in the TME facilitates increased tumor growth and metastasis, eventually leading to reduced response to ICIs [121]. CXCR1/2 inhibitors showed enhanced infiltration of CD8+ T cells, thereby reducing the entry of TANs in TME, and improving ICIs‐mediated cytotoxicity. One such CXCR1/2 inhibitor, combined with pembrolizumab, SX‐682, is being tested for stage III/IV metastatic or recurrent NSCLC [123]. Tumor‐associated macrophages (TAMs) are potential targets for ICIs therapy. In this category, therapeutics targeting CD47 and LILRB2 are in early development stages [124]. The CD47/SIRP‐alpha inhibitory axis plays a role in regulating tumor cell phagocytosis by macrophages, while LILRB2 negatively regulates the activation of myeloid cells [124]. NK cells are another target for ICIs‐based therapies. Higher NK cell infiltration in TME correlates with enhanced cell‐based cytotoxicity and better prognosis. CD8+ T cells and NK cells express NKG2A as an inhibitory receptor, which, upon binding to HLA‐E (a type of non‐classical MHC I) suppresses CD8+ T cell and NK cell function [125]. One promising therapeutic, monalizumab, which targets NKG2A, has shown encouraging results in clinical trials for advanced NSCLC [125, 126]. Other NK‐cell activating and inhibitory receptors, such as NKp46, CD16, and KIR3DL2, may serve as future targets.

### 5.2 Combination Strategies With Reprogrammed Tregs

Regulatory T cells (Tregs) produce immunoregulatory cytokines, such as IL‐10, by expressing CTLA‐4 on their surface, and infiltration of Tregs in solid TME is positively associated with poor prognosis [1]. Tregs bind to IL‐2 with high affinity, resulting in less availability of IL‐2 to effector T cells, which leads to reduced effector T cell activity, and CTLA‐4 blockade lowers Tregs by increasing effector CD4+ and CD8+ T cells [128]. The dominance of Tregs creates a TGF‐β‐enriched environment, which plays a key role in increasing resistance to ICIs, promoting metastasis, and worsening prognosis [129]. TGF‐beta inhibitors, such as vactosertib and bintrafusp, are under development [130].

### 5.3 Combination Strategies With Angiogenesis Inhibitors

Targeting angiogenesis results in reduced intratumoral hypoxia while improving the TME with enhanced effector cell infiltration to prolong survival [131]. Targeting major angiogenic factors, such as vascular endothelial growth factor receptor (VEGFR), with tyrosine phosphorylation inhibitors, is a significant focus. Anti‐VEGFR treatment improved the TME by increasing neutrophil infiltration and dendritic cell (DC) maturation while decreasing levels of MDSCs and Tregs [132]. Currently, several clinical trials combining VEGF inhibitors with anti‐PD1/PD‐L1 are ongoing and have shown promising outcomes in melanoma subtypes, including mucosal and acral melanoma [2]. The VEGFR‐inhibitor axitinib, when combined with immune checkpoint inhibitors (ICIs), such as toripalimab and anti‐LAG3 agents, significantly increased CD8+ T cell activity in mucosal melanoma and advanced renal cell carcinoma (RCC) [132, 133]. Multiple VEGF and fibroblast growth factor (FGF) receptor inhibitors, combined with lenvatinib and anti‐PD1, have enhanced CD8+ T cell activity and decreased tumor‐associated macrophages (TAMs), thereby prolonging survival in RCC, liver, and endometrial cancers which is further under study in several solid tumors, including head and neck cancer, cholangiocarcinoma, gastro‐esophageal adenocarcinoma, and ovarian cancer [135]. A trial of nivolumab‐axitinib combination assessed hypoxic cells as a biomarker to evaluate treatment efficacy [134]. Additionally, some studies utilize tumor molecular profiling to select patients for tailored combination therapies involving ICIs and VEGFR inhibitors, demonstrating favorable clinical outcomes [136]. Implementing biomarker‐driven strategies in future clinical studies will improve the accuracy of predicting treatment responses and enhance overall efficacy.

Approaches with the strongest translational momentum in immunosuppressed TMEs are combinations that pair ICIs with anti‐angiogenic agents, which have demonstrated phase‐III survival benefit and shown mechanistic evidence of TME reprogramming, such as vascular normalization, reduced MDSCs/Tregs/M2‐like macrophages, and increased dendritic and cytotoxic T‐cell infiltration [136, 137, 138]. Although strategies targeting MDSC or Treg reprogramming remain biologically compelling, they are largely confined to early‐phase development and should advance cautiously, supported by rigorous intratumoral immune profiling and phased toxicity evaluation.

## 6 Targeting Cold TME

Besides altering the tumor's natural environment, directly introducing engineered external immune cells or cellular components to boost tumor antigen‐specific immune cells, known as adoptive cell therapy, is a promising research area. Giving exogenous cells can be helpful for selectively expanding and improving the effector immune cell response [1].

### 6.1 Combination Strategies With Tumor‐Infiltrating Lymphocytes Therapy

Higher infiltration of effector immune cells in TME is positively linked to improved TME conditions and better prognosis in multiple cancer types [140]. Although tumor antigen‐specific TIL administration alone had limited success, combination approaches, such as adjusting the TME before and after TIL therapy, are being studied for enhanced effectiveness [141]. Using chemotherapies, such as fludarabine/cyclophosphamide and radiotherapy prior to TIL treatment has shown improved outcomes [142]. TIL administration followed by IL‐2 infusion can promote the expansion of the transplanted TILs; however, it may also cause capillary leakage and flu‐like symptoms [1]. In early trials of autologous TIL therapy, 19 out of 93 patients exhibited durable responses, particularly those with melanoma [143]. Additionally, a phase 2 trial combining TIL therapy with pembrolizumab demonstrated sustained responses and increased efficacy in melanoma [144]. Currently, lifileucel was recently FDA‐approved in February 2024 as the first TIL therapy for patients with advanced melanoma who previously received anti‐PD1 therapy and BRAF inhibitors if BRAF‐positive [17]. Lifileucel is also being evaluated in immunotherapy‐resistant NSCLC, with promising early results [145]. Recently, selected‐TIL therapy showed better clinical responses with anti‐PD1 in MMR‐proficient GI cancers that were unresponsive to unselected or bulk TIL therapy [146]. These findings offer new hope for patients traditionally considered unresponsive to standard TIL treatments.

### 6.2 Combination Strategies With CAR‐T Therapy

CAR‐T therapy involves collecting T cells from the patient's peripheral blood through apheresis, followed by selection, engineering, and re‐administration of the engineered T cells after a preparative treatment [1]. Recent advances have enabled CAR‐T cells to respond to repeated antigen exposure because of various co‐stimulatory domains (CD28, ICOS, OX‐40, 4‐1BB) included in CAR‐T constructs [147]. Six CAR‐T therapies targeting either BCMA or CD‐19, two B cell antigens, have been FDA‐approved for certain hematologic cancers such as lymphomas, some types of leukemia, and multiple myeloma [148]. In a phase I trial (NCT02414269), pembrolizumab combined with mesothelin‐targeted CAR‐T cells showed a median overall survival of 23.9 months and improved persistence of CAR‐T cells in malignant pleural mesothelioma [149]. The lisocabtagene maraleucel CAR‐T therapy was approved by the FDA in 2024 for mantle cell lymphoma and, in December 2025, received a new indication for marginal‐zone lymphoma (MZL), marking it as the first U.S.‐approved CAR‐T therapy for MZL [149, 150]. Although adding more co‐stimulatory molecules to CAR‐T improves efficacy and durability, creating a more immunogenic environment can cause toxicities like cytokine storm and neurotoxicity, leading to CAR‐T therapy‐related deaths of up to 15%. These risks can be mitigated by combining treatments with immunomodulators [152]. Progress in early diagnosis and toxicity management may improve patient outcomes [152]. Additionally, the lengthy manufacturing process and inconsistent success rates in producing CAR‐T cells underscore the need for a standardized approach to CAR‐T manufacturing.

### 6.3 Combination Strategies With T Cell Receptor (TCR) Therapy

T cell receptor therapy involves engineering the T cell receptor and expanding it ex vivo, then readministering it into patients so that the potential immune response against the tumor can be enhanced due to the alteration in the T cell component [1]. While CAR‐T cells can recognize potential antigens in the absence of MHC, TCRs need antigens presented by MHC to recognize [1]. Thus, MHC downregulation‐mediated immune tolerance may occur in TCR therapy, while in CAR‐T, it doesn't [153]. While CAR‐T can only respond to antigens expressed on the membrane, TCRs can recognize antigens expressed both extracellularly and intracellularly, even in low numbers [147]. One advantage of TCR therapy over CAR‐T is the ability to engineer the TCR to respond to antigenic heterogeneity, but target recognition has been challenging due to on‐target and off‐target toxicities, especially if the on‐target epitopes are also present off‐target in normal tissues, as reported in the clinical trial of anti‐MAGE‐A3 TCR therapy [154]. Despite life‐threatening toxicities, the response rate in early clinical trials is promising; for example, the phase 1/2 trial of TCR therapy targeting NY‐ESO‐1 resulted in an 80% response rate [155]. Finding highly specific epitopes of tumor‐specific antigens or viral markers can enhance drug efficacy while minimizing off‐target toxicities [1]. Combining TCR therapy with T cell agonists significantly enhances anti‐tumor efficacy by overcoming inhibitory signals in the tumor microenvironment and boosting T‐cell survival and function, as demonstrated in multiple clinical trials across various cancer types, with T‐cell agonists applied either as standalone treatments or together with immune checkpoint blockade [156]. This synergistic approach improves the therapeutic potential of TCR therapies, leading to better treatment outcomes.

Adoptive cell therapies, most notably FDA‐approved CAR‐T therapies, show the greatest near‐term clinical maturity in hematologic malignancies and, more recently, in solid tumors with the FDA approval of a TIL therapy and TCR‐based bispecific agent [156, 157]. For cold tumors, priority should be placed on rigorously controlled combination trials that integrate adoptive cell therapies with ICIs only after establishing antigen fidelity, preserved antigen presentation machinery, and acceptable additive toxicity.

## 7 Targeting Non‐Immunogenic Tumors to Enhance the Production of Tumor‐Specific Antigens

### 7.1 Combination Strategies With Chemotherapy

Chemotherapeutic agents that kill cancer cells generate tumor‐derived antigens, which can trigger a strong, vaccination‐like immune response within the tumor environment. This promotes immunogen‐mediated cell death as well as antigen processing and presentation machinery [1, 2]. Drugs such as 5‐fluorouracil, gemcitabine, and arguably cyclophosphamide, enhance the immunosuppressive TME by increasing tumor‐specific effector T cells while decreasing regulatory T cells and MDSCs, making these chemotherapies promising candidates for combination with immunotherapies [158, 159, 160]. Gemcitabine and cyclophosphamide also support cross‐presentation of antigens and the cross‐priming of effector T cells targeting tumors [1, 2]. Patients receiving cyclophosphamide prior to T cell transfer therapy and oncolytic virus therapy experience reduced immunosuppressive cells and enhanced priming of tumor‐specific effector T cells [1]. Furthermore, combining cyclophosphamide, doxorubicin, and vincristine with immunotherapy promotes M1 polarization of tumor‐associated macrophages, thereby boosting anti‐tumor immune responses [162]. The FDA approved carboplatin and pemetrexed in combination with pembrolizumab as a first‐line immunotherapy for NSCLC [163]. Platinum‐based chemotherapies paired with multiple ICIs, such as tislelizumab, nivolumab, pembrolizumab, durvalumab, and amivantamab, have shown improved survival benefits and were recently approved for treating advanced esophageal squamous cancer, NSCLC, and endometrial cancer [163, 164]. Similarly, paclitaxel was approved by the FDA for use with atezolizumab to treat TNBC, and with pembrolizumab/durvalumab for endometrial cancer, resulting in prolonged survival benefits [165, 166, 167]. The effectiveness of these combination strategies can be further enhanced by understanding their mechanisms of action and optimizing dosage and sequencing.

### 7.2 Combination Strategies With Radiotherapy

Radiation induces tumor‐specific immunity by killing the tumor cells and releasing antigens specific to the tumors [1]. These tumor‐specific antigens are damaged DNA from radiotherapy, which enhances MHC‐I expression and increases antigen presentation by macrophages. This process results in decreased CD47 and increased release of reactive oxygen species (ROS) and pro‐inflammatory cytokines, leading to greater infiltration of effector T cells [81]. Radiation also stimulates a systemic anti‐cancer immune response through a bystander effect in tumors distant from the irradiated site, causing tumor regression [1]. This abscopal effect was observed in melanoma patients treated with anti‐CTLA4 and radiation [169]. Additionally, combining anti‐PD1 therapy with radiotherapy improved survival in NSCLC [170]. In a phase II clinical trial, patients with cutaneous melanoma showed better responses when oncolytic virus immunotherapy (T‐vec) was combined with radiation [171]. Success in preclinical studies has resulted in FDA approval of chemoradiotherapy combined with durvalumab for treating stage‐III NSCLC [172]. Mechanistically, radiotherapy‐damaged DNA activates STING signaling to boost type‐I IFN production [2]. Combining STING signaling agonists, such as MV‐626, with radiotherapy enhances T cell‐mediated anti‐tumor immunity to prolong OS, and the combination of radiation, STING agonist TAK‐676, and pembrolizumab to treat patients with advanced NSCLC, triple‐negative breast cancer (TNBC), and squamous cell carcinoma of the head and neck (SCCHN) [173]. However, while radiation provides benefits, it can also have immunosuppressive effects, such as increased MDSCs, Tregs, and TGFβ in TME), along with reduced infiltration of CD8+ T cells [81]. This highlights the importance of optimizing the radiation dose and sequence, and combining it with other strategies like chemotherapy and immunotherapy to mitigate immunosuppression and improve outcomes [174].

### 7.3 Combination Strategies With Targeted Therapy

Targeted therapy effectively modulates tumor immunity, thereby reducing tumor burden; however, the challenge of developing drug resistance remains. This issue can be addressed by exploring how the tumor microenvironment (TME) is modified by targeted therapies and investigating effective combination strategies with immunotherapy [1]. Targeted therapies with superior anti‐cancer effects, such as combined inhibitors of BRAF and MEK, also increase the production of tumor‐specific antigens and MHC expression in melanoma. This leads to greater infiltration of tumor‐specific effector T cells and increased release of pro‐inflammatory cytokines by boosting the infiltration of antigen‐presenting cells and reducing VEGF levels [175]. In patients with advanced renal cancer, treatments like axitinib‐pembrolizumab and axitinib‐avelumab significantly prolong overall survival (OS) and progression‐free survival (PFS), and improve objective response rate (ORR), earning FDA approvals [176]. Additionally, lenvatinib combined with pembrolizumab has been FDA‐approved for treating advanced renal and endometrial cancers [177]. The BRAF inhibitor also depletes immunosuppressive cells such as regulatory T cells and MDSCs [1]. The temporal nature of these immune‐modulatory effects of BRAF and MEK inhibitors can be enhanced through sequential administration approaches [178]. Recent interest has grown in combining immunotherapies with targeted therapies that have promising anti‐tumor effects. For example, anti‐PD1 agents like pembrolizumab and spartalizumab, when combined with dabrafenib‐trametinib, have improved PFS in melanoma patients with BRAF‐V600E/K mutations [179]. Clinical trials combining anti‐PD‐L1 agents, such as Avelumab, with talazoparib have also yielded durable clinical benefits in patients with BRCA1 and BRCA2 alterations [180]. Furthermore, treatments that upregulate PD‐L1, such as targeted therapy for HER2, enhance antigen presentation and improve the immunogenicity of the TME, thereby activating both innate and adaptive immune responses. Ongoing clinical trials are exploring combinations of HER2‐targeted therapies with immune checkpoint inhibitors for various cancer subtypes [181]. Despite these successes, it is crucial to address the increased risk of toxic side effects, such as hepatotoxicity, associated with these combination treatments to ensure safer clinical outcomes.

### 7.4 Combination Strategies With Small‐Molecule‐Based Targeted Therapy

In melanoma, approximately 40% of patients carry different BRAF mutations, for which combined BRAF and MEK inhibitors achieve an ORR of 60%–70% [2]. However, ICIs, despite offering lower ORR regardless of BRAF mutation status, provide greater durability. This led to the combination of ICIs with BRAF/MEK inhibitors to improve therapeutic outcomes. Preclinical studies have shown that anti‐PD1/PD‐L1 therapies combined with targeted treatments promote T cell infiltration, antigen expression, and favorable TME modulation, including reduced MDSC activity and restored dendritic cell function [181, 182]. However, MEK inhibition may impair T cell activity, and BRAF/MEK inhibition can hinder dendritic cell maturation and T cell activation, complicating triplet therapy [184]. Three major clinical studies tested anti‐PD‐L1 with BRAF/MEK inhibitors in melanoma, but only one received approval due to toxicity [2, 184]. Toxicity concerns and the superior efficacy of immune checkpoint inhibition have limited the use of triplet combinations as frontline treatments; nonetheless, the Phase II TRICOTEL trial demonstrated the intracranial efficacy of triplet therapy for BRAF(V600E/K)‐mutant melanoma [186]. In the ongoing S2000 trial, the combination of nivolumab with encorafenib and binimetinib showed longer PFS compared to ipilimumab plus nivolumab in patients with symptomatic melanoma with brain metastases [187]. Toxicity was observed with both combination types; however, it was relatively lower in the triplet setting [187]. Addressing toxicity is a critical factor in designing combination therapies that involve small‐molecule inhibitors and immune checkpoint inhibitors.

Chemotherapy and radiotherapy combinations with ICIs already have Phase‐III evidence in multiple settings and should remain top priorities for near‐term implementation, with attention to regimen‐specific immunogenicity and lymphodepleting risk [188]. Targeted therapy combinations, including anti‐VEGF with ICIs, are another high‐priority research area when there is strong disease‐specific Phase‐III or approval data [137]. Small‐molecule combinations require careful pharmacokinetic/pharmacodynamic and toxicity evaluation before large‐scale testing.

## 8 Lessons From Failed Clinical Trials

Translating promising preclinical combination strategies into positive phase‐III outcomes has proven harder than anticipated. A careful analysis of representative negative phase III studies, such as the IDO1 inhibitor epacadostat plus pembrolizumab (ECHO‐301/KEYNOTE‐252), SKYSCRAPER trials of anti‐TIGIT tiragolumab, and randomized studies of oncolytic virus (T‐VEC) plus anti‐PD1, highlights the following recurring failure modes: (1) inadequate demonstration mechanism of action at the doses used; (2) insufficient biomarker‐driven patient selection; (3) suboptimal drug sequencing, and (4) overreliance on preclinical models that do not recapitulate human TME complexity. The ECHO‐301 epacadostat–pembrolizumab trial revealed uncertainties about IDO1 target inhibition and compensatory metabolic escape pathways, arguing for rigorous tumor pharmacodynamics and pharmacokinetic/pharmacodynamic readouts in future metabolic immunotherapy trials [188, 189]. Trials that often advance without clear demonstration that the investigational agent modulates its intended pathway in human tumors at clinical doses, should mandate tumor pharmacodynamic readouts to confirm the mechanism of action early [188, 189]. Similarly, the SKYSCRAPER tiragolumab trial underscores the importance of prospective biomarker enrichment [58]. Heterogeneous expression of targets, such as variable co‐expression of PD‐L1 and alternative checkpoints, can dilute signals in broad, unselected Phase‐III populations, thus prospective enrichment and validated companion diagnostics are frequently required. Overinterpretation of small Phase‐II signals without robust biomarker or mechanistic concordance has led to Phase‐III failures [191], thus larger or randomized Phase‐II trials with embedded biomarkers provide more reliable data. Another randomized phase III trial of T‐VEC with anti‐PD1 combination suggests that intratumoral delivery limitations, patient selection, and sequencing may reduce benefits in unselected patients [109]. Trials should prospectively define which patients are likely to benefit and optimize timing relative to systemic ICIs. Synergy in preclinical models can be highly sequence‐dependent, yet many trials test concurrent administration without prior optimization. Moreover, pharmacokinetic/pharmacodynamic mismatches or antagonistic scheduling can erase efficacy [192]. These failures do not invalidate the biological rationale behind combinations, but they do demand more rigorous early translational work, prospective biomarker strategies, and trial designs that explicitly test mechanistic assumptions. Common murine models often fail to replicate the human TME complexity, leading to overestimation of efficacy [191]. Incorporating humanized, organotypic, or multi‐modal preclinical validation and early human pharmacodynamic studies reduces translational risk.

## 9 Toxicity in Combination Therapy

Combining immunotherapies with each other or with cytotoxic, targeted, or cell‐based modalities increases both the incidence and complexity of adverse events compared to single agents. Below, we summarize overlapping versus unique toxicities by modality, provide quantitative context from recent meta‐analyses and guideline statements, outline practical mitigation strategies, propose a risk–benefit framework for trialists and clinicians, and review the most promising predictive biomarkers for immune‐related adverse events (irAEs) and treatment‐specific toxicities.

### 9.1 Overlapping Versus Unique Toxicities

Compared to monotherapy, dual‐ICI regimens substantially increase rates of high‐grade immune‐related adverse events, including colitis, hepatitis, endocrinopathies, and dermatitis. Meta‐analytic and guideline data report substantially higher grade(grade 3 to grade4) irAE rates with combination ICI regimens versus single agents, and fatal irAEs, while rare, occur and require rapid recognition and management [192, 193]. Chemotherapy can produce classic cytopenias, mucositis, and organ toxicities that overlap with or mask irAEs [192, 193]. Successful Phase‐III combinations, such as chemotherapy with pembrolizumab in NSCLC, were enabled by careful regimen selection and proactive supportive care to limit overlapping myelosuppression [194, 195]. Angiogenesis inhibitors combined with ICIs can improve immune infiltration but come with vascular toxicities, such as hypertension and thromboembolism, and hepatic or renal effects that may overlap with immune hepatitis or nephritis [196, 197]. These overlapping organ toxicities mandate rigorous run‐in safety cohorts and organ‐specific monitoring [196, 197]. CAR‐T and some T‐cell engager therapies carry risks of cytokine release syndrome (CRS) and immune effector cell‐associated neurotoxicity syndrome (ICANS), which can be life‐threatening and require specialized management. Combining these agents with ICIs risks enhancing systemic immune activation and potentially amplifying systemic toxicity; therefore, dose‐finding and conservative sequencing are recommended [198, 199, 200, 201].

### 9.2 Quantitative Contexts and Notable Figures

A pooled meta‐analysis of fatal toxic effects associated with ICIs reported that treatment‐related deaths are uncommon but measurable, with organ‐specific fatality patterns, such as myocarditis and pneumonitis. This highlights the need for vigilance and early intervention in combination regimens [194]. For adoptive therapies, systematic reviews summarizing CAR‐T trials through 2022 report non‐relapse mortality estimates on the range of ∼ 4% to 7%, and CRS/ICANS remain primary early toxicities requiring institution‐level readiness [199, 202]. These estimates contextualize both clinical risk and the need for risk evaluation and mitigation strategies‐like operational preparedness during trials.

### 9.3 Practical Mitigation Strategies

***Early recognition and triage***: Standardized toxicity grading and rapid triage pathways, such as CTCAE grading; institution‐specific escalation to ICU, are foundational. Use of published management algorithms for irAEs and CRS/ICANS is mandatory for trial sites [192, 198].

***Pharmacologic interventions***: Corticosteroids remain the mainstay for most high‐grade irAEs, where early initiation is associated with reversal of many immune toxicities [193]. Corticosteroids are also used for steroid‐refractory cases and ICANS management. IL‐6 blockade, such as tocilizumab is the standard for moderate to severe CRS and is widely recommended by consensus guidelines. Institutional availability of tocilizumab and clear on‐site protocols significantly improve safety [195, 198]. Organ‐specific measures, such as mycophenolate or infliximab for steroid‐refractory hepatitis/colitis should be pre‐specified in protocols [193].

### 9.4 Trial Design Safeguards

Safety run‐in cohorts with prespecified stopping rules for overlapping toxicities, particularly in first‐in‐human combination dosing, are essential for minimizing early risk [198]. Adaptive trial designs that enable early termination for unacceptable toxicity or permit the sequential addition of optimized sequencing or dosing cohorts can further mitigate the likelihood of late‐stage failures [198]. For larger combination studies, centralized adverse‐event adjudication and oversight by an independent Data and Safety Monitoring Board (DSMB) are strongly recommended to ensure consistent and rigorous safety evaluation [193].

### 9.5 Framework for Assessing Risk–Benefit in Combinations

For each proposed combination, prospective documentation and justification of the following items is recommended in the protocol: (1) biologic rationale for synergy with explicit mechanistic pharmacodynamic endpoints to test, such as intratumoral T cell infiltration, and cytokine changes [195, 203], (2) expected overlapping toxicities and management algorithms, such as drug choice, dosing, and timetables [193], (3) safety run‐in design and stopping rules, such as dose‐limiting toxicities and thresholds for cohort expansion or pause [198], (4) operational preparedness, such as availability of tocilizumab/steroids/ICU beds, staff training on CRS/ICANS or severe irAEs [198], and (5) translational biomarker plan for both efficacy and toxicity, such as serial ctDNA, cytokines, autoantibodies, and on‐treatment biopsies when possible [205].

### 9.6 Emerging Predictive Biomarkers for Adverse Events

Although validated clinical predictors of irAEs remain an active area of investigation, several candidate biomarkers show promise and may be incorporated into prospective studies.

***Baseline autoantibodies and pre‐existing autoimmunity***: Presence of autoimmune serologies at baseline has been associated with higher risk of irAEs in multiple cohorts and may predict organ‐specific events, such as thyroiditis, and prospective validation is ongoing [197, 204].

***Peripheral blood immune signatures***: Early declines in circulating B‐cell subsets, elevations in specific cytokines (IL‐6, IL‐17), and baseline high neutrophil‐to‐lymphocyte ratio have been associated with higher irAE risk. These markers are attractive because they are minimally invasive and repeatable [205, 206].

***Germline HLA and genetic variants***: Certain HLA alleles, such as HLA‐A*03, and germline variants have been implicated in increased irAE susceptibility. Larger multi‐center cohorts are needed for validation across tumor types [206, 207].

***Multi‐omic approaches***: Integrative models that combine baseline autoantibodies, cytokine panels, germline markers, and early on‐treatment dynamics, such as ctDNA decrease, appear most promising for robust prediction and are an active focus of recent high‐impact work. Prospective incorporation into trials is recommended [197, 204].

## 10 Leveraging Next‐Generation Strategies for Early Biomarker Discovery to Predict Immunotherapy Response

**FIGURE 4:**
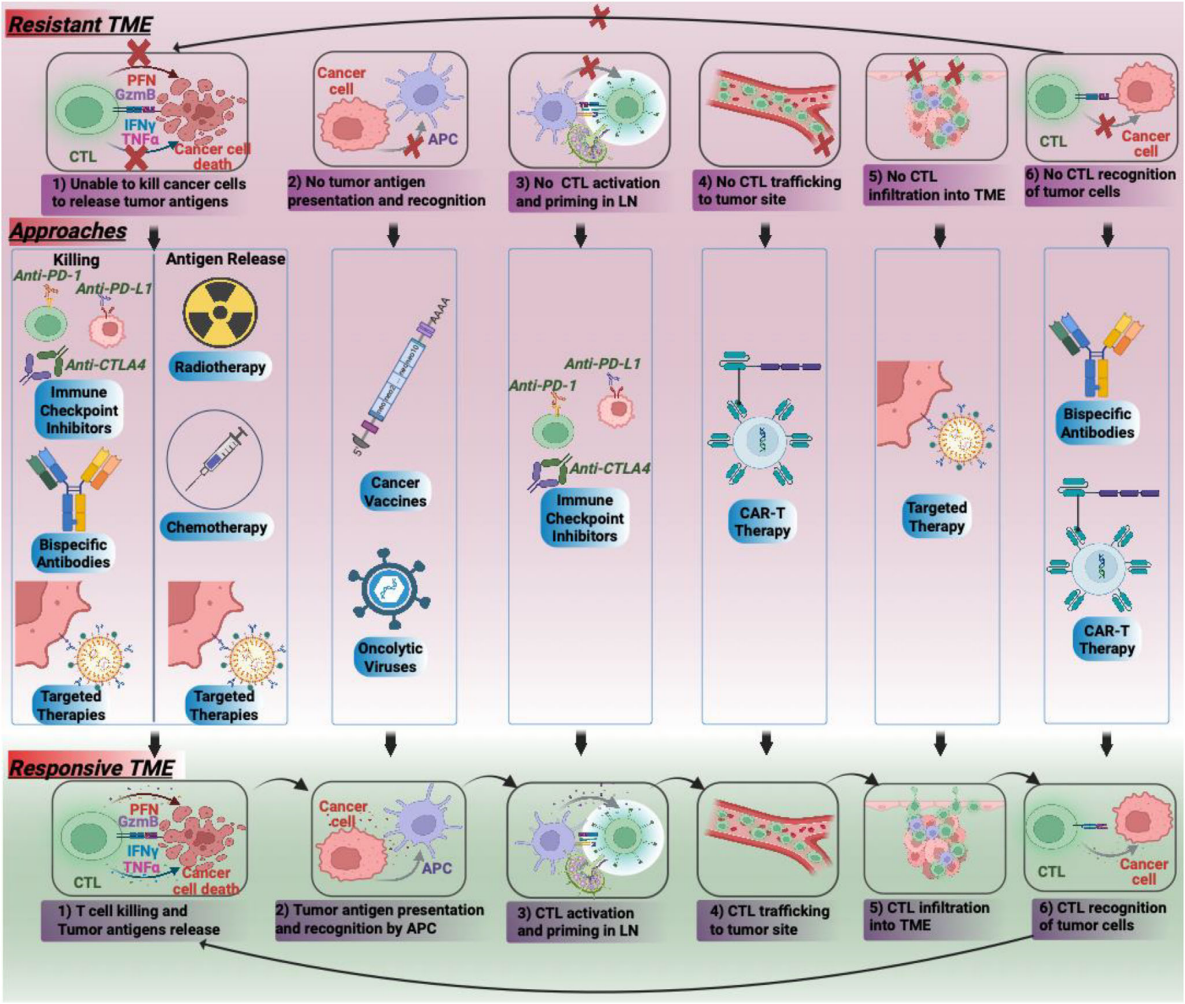
Therapeutic approaches targeting different stages of the tumor immunity cycle to sensitize resistant TME to ICIs. Implementing combination strategies, along with ICIs, to target the particular stage in the cancer immunity cycle enhances the response to ICIs. Created in BioRender. Mahmanzar, M. (2025) https://BioRender.com/dzb508x.

Higher tumor mutational burden (TMB), higher PD‐L1 expression, microsatellite instability‐High (MSI‐H), and recently lower ctDNA levels are FDA‐approved biomarkers of response to ICIs. Determining the conventional biomarkers, especially PD‐L1 expression, is challenging due to the lack of standard assays, variable threshold cutoffs, and low‐expression responders [3]. Therefore, a deeper understanding of the individual TME is crucial to address this challenge caused by tumor heterogeneity [2, 25]. To overcome these challenges, next‐generation predictive biomarkers, such as serial ctDNA measurement, are widely considered, alongside managing ICIs‐mediated toxicities (Table 2). In addition to FDA‐approved biomarkers, other biomarkers, such as VEGF, T cell activation/exhaustion markers, immunosuppression markers, tumor‐specific neoantigens, tumoral soluble factors, epitope breadth, tertiary lymphoid organs, microbiome, HLA genotype, metabolic markers, cellular neighborhood, methylation markers, and autoantibodies are currently under investigation to predict the response to ICIs (Table 2). Efforts are focused on targeting the innate immune system and specific stages of the cancer‐immunity cycle (Figure 4). For example, combining anti‐CTLA4 and immunosuppression modulators with cancer vaccines can boost the expansion and maturation of vaccine‐induced effector cells and improve immune cell trafficking into the TME [1]. However, limitations of preclinical studies, such as lack of treatment histories, patient‐specific TME variations, routine tumor monitoring, and systemic effects of tumors on immunity, are critical factors to consider in developing combination immunotherapies.

**TABLE 2:** FDA‐Approved and Investigational Predictive Biomarkers: Conventional, Emerging, and Next‐Generation Approaches for combination with existing ICIs.

Conventional Biomarkers	Prediction	Site specification for biomarker assessment	Investigated malignancies and clinical indications for biomarker	Biomarker assessment methodologies	Combination therapeutic strategies explored	Clinical outcomes and treatment efficacy	Biomarker advantages and utility	Biomarker limitations and challenges
**PD‐L1 high [** 3, 233, 234, 235, 236]	Response	Tumor cells, immune cells in the TME	FDA‐approved: NSCLC, gastroesophageal cancers, cervical cancer, urothelial cancer, HNSCC, TNBC Phase I‐III trials: multiple cancer types	IHC (22C3 Dako, SP263 Ventana, 28‐8 Dako), scored based on the Tumor Proportion Score (TPS) or Combined Positive Score (CPS)	Starting with pembrolizumab and nivolumab, evolved into a companion diagnostic for atezolizumab and durvalumab	Improved response rates and PFS, better outcomes observed in NSCLC and melanoma	Clinically validated and standardized for use with FDA‐approved therapies, Provides actionable insights for treatment selection	High heterogeneity within tumors leads to inconsistent results, Variability across different IHC assays complicates standardization
**VEGF [** 132, 133, 135, 237, 238, 239, 240, 241, 242, 243, 244]	Resistance	Tumor tissue, blood	FDA‐approved: None Phase I‐III trials: Advanced RCC, HCC, endometrial cancer, and other cancers	ELISA, IHC, PCR	Nivolumab‐axitinib and hypoxic cells, ICIs‐a‐VEGF and tumor molecular profile, lenvatinib‐ICIs	Enhanced anti‐tumor response with good safety performance, improved PFS and OS	VEGF's central role in angiogenesis makes it a viable target for therapeutic intervention, with several approved agents demonstrating clinical benefit	Lack of Standardization for VEGF levels, Tumors may develop resistance to VEGF‐targeted therapies through alternative angiogenic pathways
**CD8+ TIL [** 245, 246, 247]	Response	TME	FDA‐approved: None Phase I‐II trials: Melanoma, NSCLC, urothelial cancer, and other cancers	IHC	ICIs; UI: adoptive cell therapies	Improved clinical outcomes in advanced melanoma, and high CD8 density correlates with improved survival and response to ICIs	CD8+ TIL is a robust indicator of immune response	It does not account for the functional state of T cells, limiting predictive power
**IDO1 [** 248, 249, 250, 251]	Resistance	Tumor tissue	FDA‐approved: None Phase I‐III trials: Melanoma, NSCLC, head and neck cancer, urothellial cancer, ovarian cancer, glioma, sarcoma	IHC, qPCR	Epacadostat with pembrolizumab and nivolumab	Mixed outcomes	IDO1 reflects metabolic immunosuppression, enabling combination therapies targeting immunosuppression pathways	IDO inhibition alone may be insufficient for therapeutic efficacy, highlighting the need for combination strategies
**FOXP3+ TILs [** 252, 253, 254]	Resistance	Tumor tissue, TME	FDA‐approved: None Phase I‐II trials: Melanoma, ovarian cancer, hepatobiliary cancer	IHC, qPCR	Used to guide therapies targeting regulatory T cell suppression, such as anti‐CTLA‐4 combinations	Delayed tumor growth and reduced metastasis	Potential biomarker for identifying patients who may benefit from therapies targeting immunosuppressive pathways	The dual role of FOXP3 in immune tolerance and tumor immune evasion presents challenges as systemic depletion of FOXP3+ Tregs may lead to autoimmunity
**High Tumor Mutational Burden High (TMB‐H) [** 255, 256, 257, 258]	Response	Tumor tissue	FDA‐approved: Pembrolizumab for TMB‐H (≥10 mut/Mb) solid tumors Phase I‐III trials: NSCLC, melanoma, urothelial cancer, colorectal cancer, breast cancer, HNSCC, cervical and ovarian cancers, multiple other cancers	PCR, NGS; Emerging: plasma‐based NGS	Pembrolizumab and TMB‐H for solid tumors; UI: TMB with a‐LAG3, TMB with a‐TGIT	Improved responses to pembrolizumab	Independent of PD‐L1 expression to predict response to ICIs, expanding patient eligibility	Variability in TMB assessment methods and cutoff values can affect consistency in patient selection
**MSI‐H/ dMMR and/or co‐mutation with HRR and BER [** 245, 259, 260]	Response	Tumor tissue	FDA‐approved: Solid tumors including colorectal cancer Phase I‐III trials: CRC, endometrial cancer, GC, multiple MSI‐H solid tumors	WES, Targeted NGS Panels (FoundationOne CDx); Emerging: ctDNA analysis	MSI‐H with pembrolizumab, MSI‐H with pembrolizumab and Nivolumab, MSI‐H with nivolumab and ipilimumab; UI: MSI‐H with CAR‐T therapy	Enhances neoantigen load, Durable response, Enhanced ORR, PFS, and OS with co‐mutation	Highly predictive of anti‐PD1 response, ctDNA‐based assays may allow less invasive monitoring	A tiny population (∼4%) of tumors are MSI‐H, heterogeneity within tumors may lead to sampling errors
**Neoantigens resulting from tumor‐specific mutations [** 261, 262]	Response	Tumor tissue, ctDNA in blood	FDA‐approved: None Phase I‐III trials: Melanoma and multiple other cancers	NGS, MS, In Silico Prediction Models, T Cell reactivity assays to determine immunogenicity of neoantigens	Neoantigen vaccines in combination with ICIs, personalized cancer vaccines, Adoptive T cell therapy, neoantigen‐pulsed DC vaccine	Improved ICIs response, demonstrated safety and immunogenicity in early trials	Neoantigens are unique to tumor cells, reducing the risk of targeting healthy tissues, allows for tailored immunotherapies based on individual tumor profiles	Inter‐ and intra‐tumoral heterogeneity, accurate detection of neoantigens requires sophisticated sequencing and bioinformatics tools
**IFN**γ [35, 263, 264, 265, 266]	Variable ‐Response ‐Resistance (Defective or chronic low‐dose exposure of IFNγ production)	Tumor tissue	FDA‐approved: None Phase I‐III trials: Melanoma, NSCLC, HNSCC, GC, urothelial cancer, and multiple solid cancers	qRT‐qPCR, FC, nanostring TIS, IHC; Emerging: RNA seq, liquid biopsy, CyTOF, RNA‐ISH	High IFNg as a biomarker of response to nivolumab and pembrolizumab Emerging: TIL therapy, combination, and gene therapies	Predicts better ORR, PFS, and OS with anti‐PD1 therapies, sustained expression induces resistance to anti‐PD1	Robust predictor of response in hot TME	Heterogenous expression, high expression leads to immunosuppressive feedback leading to therapy resistance, lack of universal thresholds
**Tregs level [** 267, 268, 269]	Resistance	Tumor tissue, TILs	FDA‐approved: None Phase I‐II trials: Melanoma, hepatobiliary and ovarian cancer	FC, IHC; Emerging: RNA seq, multiplex IHC	Targeted indirectly by a‐CTLA4 and anti‐PD‐1 therapies; Emerging: low‐dose cyclophosphamide to deplete Tregs, anti‐CD25 mabs	Improved OS and PFS in ICIs‐treated patients; UI: Treg depletion therapies show promise in pre‐clinical models but limited success in human trials due to off‐target effects	Well‐established role in immune suppression and response to ICIs; Emerging: High prognostic value when combined with other markers like PD‐L1 or CD8+ T cells	high variability across different tumors, depletion strategies risk inducing autoimmunity due to non‐specific targeting
**MDSCs level** [271]	Resistance	Tumor tissue, peripheral blood	FDA‐approved: None Phase I‐II trials: Pancreatic cancer, prostate cancer, HCC	FC, IHC, ELISA; Emerging: scRNA seq	Inhibitors of CSF1R, arginase are used to reduce MDSC levels and combine with ICIs	Reduced MDSC levels are associated with better responses to ICIs	Strong predictor of immunosuppression and immunotherapy resistance	lack of standardization for marker panels and cutoffs
**T cells level specific to tumor neoantigens [** 271, 272, 273]	Response	Peripheral blood, tumor tissue, draining lymph nodes	FDA‐approved: None Phase I‐II trials: melanoma, lung cancer, ovarian cancer	TCR sequencing, pMHC tetramer staining, sc‐RNA seq; Emerging: CyTOF, mIF	ICIs, TIL‐based adoptive T cell therapy (lifileucel), CD19‐targeting CAR‐T therapies; Emerging: neoantigen vaccines, TCR‐T cell therapies	Durable tumor regression and prolonged survival	Targeted recognition and killing of cancer cells minimizing normal cell damage, can elicit strong immune response, have personalization potential	Variability in neoantigen expression due to tumor heterogeneity affects the consistency of therapeutic response
**Anti‐tumoral soluble factors [** 274, 275]	Response	serum/plasma, tumor tissue	FDA‐approved: None Phase I‐III trials: IL‐2 for metastatic RCC and melanoma, various other cytokines for multiple other cancers	ELISA, Luminex multiplex assays, IHC, FC, qRT‐PCR; Emerging: scRNA‐seq, CyTOF, exosome‐based assays	High‐dose IL‐2 for advanced RCC and melanoma but with higher toxicity, IFN‐γ signatures with ICIs; UI: targeting IL‐6, and CXCL9/ CXCL10 in combination with ICIs	Durable response in high dose IL2 therapy, higher IFNg correlates with better response to ICIs, a‐IL6 reduces CRS‐related mortality in CAR‐T therapy	Predicts response to ICIs, accessible via non‐invasive blood sampling	Variability in cytokines levels, lack of standard assay methodologies, and higher cytokines levels also indicate systemic inflammation unrelated to cancer
**Pro‐tumoral soluble factors** [277]	Resistance	Tumor tissue, blood	FDA‐approved: None Phase II‐III trials: RCC, HCC, endometrial cancer	ELISA, Luminex multiplex assays, IHC, FC, qRT‐PCR; Emerging: scRNA‐seq, CyTOF, exosome‐based assays	Targeting VEGF‐A, TGF‐β and IL‐10 with ICIs	Reverse immunosuppressive resistance to ICIs	Guides rational design of ICIs combinations	The complexity of TME makes it difficult to attribute treatment outcomes in a single cytokine
**Body mass index [** 277, 278, 279, 280]	Response	Non‐invasive measures not specific to any particular organ	FDA‐approved: None Phase II‐III trials: Analyzed as covariate across multiple tumor types	BMI calculation using formula weight/ (height)^2^	Impact of BMI on ICIs outcome; for example, Weight‐based dosing of ICIs exhibited improved PFS and OS	Higher BMI is linked to improved OS in patients treated with ICIs	Easily measurable, Higher BMI may correlate with improved immunotherapy efficacy in certain cancers	Variability, inconsistent relationship between BMI and immunotherapy outcomes across cancer types

Emerging Biomarkers	Prediction	Site specification for biomarker assessment	Investigated Malignancies and Clinical Indications	Biomarker Assessment Methodologies	Combination Therapeutic Strategies Explored	Clinical Outcomes and Treatment Efficacy	Biomarker Advantages and Utility	Biomarker Limitations and Challenges
**Level of diversified epitopes [** 210, 281]	Response	Peripheral blood, serum, tumor tissue; Emerging: exosomal or ctDNA in the blood	FDA‐approved: None Phase I‐III trials: Advanced melanoma breast cancer, glioblastoma, and various other cancer types	ELISpot assays, FC, TCR sequencing, serology testing Emerging: scRNA‐seq	ICIs, cancer vaccines, and adoptive T cell transfer therapies promote epitope spreading	Correlates with improved therapeutic outcomes, durable tumor regression improved PFS	Indicates broad immune system activation, predicts long‐term clinical benefit in ICIs and cancer vaccines	Heterogeneity among patients, lack of standardized methods, and temporal dynamics are not fully understood
**Tertiary lymphoid organs [** 282, 283]	Response	Tumor tissue	FDA‐approved: None Phase I‐II trials: Melanoma, glioma, breast cancer	IHC, multiplex IF; Emerging: spatial transcriptomics, gene expression profiling	ICIs, adoptive cell therapy, cancer vaccines	improved prognosis, survival, and durable responses in ICIs	Indicators of active immune response and better prognosis can guide potential combination immunotherapies	Lack of standard assays and variability in composition, density, and maturation status can complicate assessment and interpretation
**Microbiome [** 284, 285, 286]	Response	Tumor, fecal samples, oral swabs	FDA‐approved: None Phase I‐II trials: Melanoma, gastrointestinal and multiple other cancers	16S rRNA sequencing, whole‐genome shotgun metagenomics, metabolomics, and functional profiling of microbiota‐derived metabolites	Emerging: fecal microbiota transplantation (FMT) in combination with ICIs	Improved response rate to ICIs when a patient exhibits favorable gut microbiome composition or undergoes FMT from responders	Can be targeted with non‐invasive ways like diet, probiotics, or FMT, offers insights into systemic immune modulation and tumor‐immune interactions.	High inter‐patient variability, lack of standardization in microbiome assessment techniques, complex interactions with immune system
**High ctDNA levels [** 219, 287]	Resistance	Plasma, other bodily fluids including CFS, urine, and plural effusions	FDA‐approved: assays for genomic profiling in multiple cancers Phase II‐III trials: NSCLC, urothelial, and multiple other cancers	NGS, ddPCR, WES	Use of ctDNA to identify MSI‐H tumors to select for ICIs treatment, ctDNA‐guided personalized cancer vaccines	Reduced ctDNA correlates with improved PFS and OS, in clinical trials, ctDNA clearance is associated with early response indicator	Non‐invasive, real‐time monitoring of tumor‐specific alterations, early response detection, broad applicability	False negatives may occur due to low levels, especially in early‐stage cancers, lack of standard protocols and interpretation, challenging cost and accessibility
**Activated and exhausted T cell phenotype** **(PD1, CTLA‐4, TIM‐3, LAG‐3, TIGIT, ICOS, CD137) [** 68, 288, 289]	Variable ‐Response (Activated) ‐Resistance (Exhausted)	Tumor tissue, peripheral blood	FDA‐approved: None Phase I‐III trials: Melanoma, NSCLC, other solid tumors and hematologic malignancies	IHC, FC; Emerging: mIHC, mIF, CyTOF, scRNA‐seq	anti‐PD1 and anti‐CTLA4, anti‐PD1 and anti‐LAG3, anti‐PD1 and anti‐TGIT, anti‐PD1 and anti‐TIM3, ICOS‐agonists with anti‐CTLA4, high CD137 with CAR‐T therapies	Durable response with anti‐PD1+anti‐CTLA4 and anti‐PD1+anti‐LAG3, anti‐PD1 with anti‐TIM3 enhanced anti‐tumor response, ICOS‐agonists with anti‐CTLA4 enhanced anti‐tumor immunity, CD137 in CAR‐T therapies correlates with treatment efficacy	A clear association of PD‐1 and CTLA‐4 expression with immunotherapy eligibility and efficacy, exhaustion markers provide deeper insights into dysfunctional T cells in non‐responders, activation markers allow tracking of early response and guide treatment optimization	Heterogenous expression within the TME and between patients, PD‐1 and CTLA‐4 expression alone may not fully predict treatment outcomes, dynamic changes in T cell phenotypes during treatment complicate interpretation and require continuous monitoring
**T cell receptor heterogeneity [** 290, 291, 292]	Response	Peripheral blood, TILs containing tumor tissue	FDA‐approved: None Phase I‐III trials: As correlate in NSCLC, HNSCC, melanoma, and multiple cancer types	High‐throughput sequencing, FC, IHC; Emerging: scRNA‐seq, spatial transcriptomics	TCR metrics with anti‐PD1, Adoptive T cell therapy therapy; Emerging: neoantigen vaccines (for inducing TCR diversity targeting tumor neoantigens)	High TCR diversity is associated with better response rates to ICIs, Clonal expansion is indicative of effective immune response	Can reflect real‐time immune status and response, directly linked to adaptive immunity and therapy response	In some of the cancers, high, may miss low‐frequency TCR clones due to limited sensitivity, which requires sophisticated analytical tools and expertise
**HLA genotype in host [** 293, 294, 295]	Variable	Peripheral blood	FDA‐approved: None Phase I‐II trials: Metastatic NSCLC, melanoma, and various other cancer types	NGS, PCR	HLA genotypes, such as HLA‐DRB4 with ICIs	Patients with the HLA‐DRB4 genotype demonstrated longer OS but increased irAEs when treated with ICIs, HLA‐A*03 allele is associated with shorter survival times post‐ICIs treatment	HLA genotyping can help tailor immunotherapy treatments based on individual genetic profiles, Useful in monitoring irAE risk	Extensive polymorphism of HLA genes complicates the identification of definitive biomarkers. Variability in genotyping methods and interpretations can lead to inconsistent results across studies

Next‐generation Biomarkers	Prediction	Site specification for biomarker assessment	Investigated Malignancies and Clinical Indications	Biomarker Assessment Methodologies	Combination Therapeutic Strategies Explored	Clinical Outcomes and Treatment Efficacy	Biomarker Advantages and Utility	Biomarker Limitations and Challenges
**Metabolic markers** (**LDH, GLUT1, IDO1, ARG1, PGC‐1α) [** 2, 245, 250, 296]	Resistance	peripheral blood, tumor tissue	FDA‐approved: None Phase I‐II trials: LDH in Melanoma, lymphoma, and others, GLUT1 in cervical cancers and others, IDO1/ARG1 in multiple cancers, including ovarian, lung, and pancreatic, PGC‐1α in various cancer types	Serum LDH assays; IHC for GLUT1; IHC, qPCR and enzyme activity assay for IDO1/ARG1; FC/WB for PGC‐1α; Emerging: MS, scRNA‐seq, metabolic PET scan	Epacadostat with anti‐PD1, ARG1 inhibitors with ICIs, targeting lactate metabolism combined with ICIs	Some studies report enhanced immune responses with anti‐IDO1 and ICIs combinations though some do not demonstrate significant clinical benefit	LDH assays are accessible and economical, GLUT1 reflects tumor metabolic activity and hypoxic status, IDO1/ARG1 indicates immunosuppression which is a potential target for immunotherapy	LDH can be elevated in conditions unrelated to cancer, metabolic inter‐ and intra‐tumoral heterogeneity complicates analysis, IHC requires invasive tissue sampling
**Cellular neighborhood (CN) [** 297, 298, 299, 300]	Variable ‐Response (TILs and PD‐L1 spatial expression) ‐Resistance (Immune‐excluded zones and immunosuppressive myeloid niches)	LN, TILs, TME	FDA‐approved: None Phase I‐III trials: NSCLC, CRC, melanoma and various other cancers	IHC, mIF; Emerging: single cell spatial transcriptomics, high‐dimensional Imaging for simultaneous analysis of multiple markers, such as CODEX	CXCL13 expression in certain neighborhoods and co‐localization of CD8+ T cells with tumor cells predicts response to ICIs, Spatial infiltration of T cells into tumors correlates with therapeutic efficacy in adoptive cell therapy	Correlates with durable responses increased antigen‐experienced T cell subsets, and reduced CCR2+ monocytes; Under investigation: improved T cell infiltration and efficacy of ICIs in CAF‐targeted therapy, Modifying hypoxic niches	Offers insights into the spatial organization of immune and tumor cells, Spatial features have been linked to responses to therapies, aiding in patient stratification	Single‐time‐point biopsy‐based spatial analysis lacks temporal resolution, The techniques are resource‐intensive, expensive, and require specialized expertise
**Methylation markers (MGMT Promoter, PD‐L1 Promoter) [** 301, 302]	Response	Tumor tissue, peripheral blood	FDA‐approved: None Phase I‐III trials: MGMT promoter methylation in glioma and glioblastoma, PD‐L1 promotor methylation in HNSCC, NSCLC, melanoma	methylation‐specific qPCR, bisulfite sequencing; Emerging: NGS, pyrosequencing	ICIs, DNMT inhibitors with immunotherapy	DNA methylation profile predicts responsiveness to ICIs, MGMT methylation is associated with improved survival	High predictive accuracy for treatment outcomes, Potential to improve prediction accuracy when combined with gene expression profiles	Lack of standardization in methylation analysis methods, variability in methylation rates reported across studies, High false‐positive rates in pan‐cancer models
**Autoantibodies (NY‐ESO‐1, MAGE‐A, against neoantigens, against anti‐PD‐1 or anti‐CTLA‐4 treatments) [** 112, 197, 303]	Response	Peripheral blood, serum, plasma, tumor tissue	FDA‐approved: None Phase I‐II trials: Autoantibodies against NY‐ESO‐1 in melanoma and sarcoma, and against MAGE‐A and NY‐ESO‐1 in TNBC	ELISA, IHC, WB, Immunome protein array	Cancer Vaccines targeting NY‐ESO‐1, ICIs with NY‐ESO‐1 targeted therapies	Vaccination with NY‐ESO‐1 peptides enhanced specific immune response, presence of certain autoantibodies increased the risk of developing irAEs during ICIs treatment	Non‐invasive sampling, Potential for early cancer diagnosis, and immune monitoring	Lack of cancer specificity, Variability in antibody production between individuals, high costs, and technical requirements

NSCLC‐ non‐small cell lung cancer; UC‐ urothelial carcinoma; TNBC‐ triple‐negative breast cancer; GC‐ gastric cancer; EC‐ esophageal cancer; HNSCC‐ head and neck squamous cell carcinoma; UI‐ under investigation; WES‐ whole exome sequencing; MS‐ mass spectrometry; NGS‐ next generation sequencing; FC‐ flow cytometry; TIS‐ tumor inflammation signature; CyTOF‐ cytometry by time of flight; RNA‐ISH‐ RNA in situ hybridization; ORR‐ objective response rate; OS‐ overall survival; PFS‐ progression free survival; pMHC‐ peptide‐bound major histocompatibility complex; mIF‐ multiplex immunofluorescence; scRNA‐ single cell RNA; PET‐ positron emission tomography; TCR‐ T cell receptor; CRS‐ cytokine release storm; FMT‐ fecal microbiota transplantation; CSF1R‐ colony stimulating factor 1 receptor; ddPCR‐ droplet digital polymerase chain reaction; irAE‐ immune‐related adverse event; HLA‐ human leukocyte antigen; LDH‐ lactose dehydrogenase; GLUT1‐ glucose transporter 1; ARG1‐ arginase 1; PGC‐1α‐ peroxisome proliferator‐activated receptor gamma coactivator 1 alpha; LN‐ lymph node; MGMT‐ O6‐methylguanine‐DNA methyltransferase; DNMT‐ DNA methyltransferase; NY‐ESO‐1‐ New York esophageal squamous cell carcinoma‐1 autoantibody; MAGE‐A‐; melanoma‐associated antigen family‐A; TILs‐ tumor infiltrating lymphocytes; MSI‐H‐ microsatellite instability‐high; dMMR‐ mismatch repair‐deficient; HRR‐ homologous recombination repair; BER‐ base excision repair; CODEX‐ co‐detection by indexing; CAF‐ cancer‐associated fibroblast.

### 10.1 Cutting‐Edge Predictive Biomarkers

### 10.2 Comprehensive Studies of Resistance Mechanisms Within the TME

Investigations into immunotherapy resistance have revealed significant vulnerabilities in current approaches, such as highly specific T cell responses that often target a single antigen or epitope. This can lead to immune editing and the development of antigen‐loss variants, like those involving EGFRVIII or B cell maturation antigen [1, 2]. Additionally, T cells exposed repeatedly and chronically to tumor antigens within the immunosuppressive TME become exhausted and lose their function [2]. The TME often lacks sufficient resources to support the metabolic needs of effector cells and antigen‐presenting cells (APCs), which further weakens immune responses [209]. Moreover, circulating effector cells may fail to reach and infiltrate the TME, decreasing their effectiveness [210]. Expanding biomarker research to thoroughly examine these resistance mechanisms is crucial for guiding the development of new combination therapies and improving patient selection strategies.

### 10.3 Expanding T Cell Response to Multiple Antigens

Epitope broadening, defined as an expanding T cell response to various antigens, has shown connections to clinical outcomes in cancer. Expanded T cell responses were associated with better results in patients treated with an MHC class I epitope‐based vaccine [211]. Similar patterns have been observed in other vaccine strategies as well as in adoptive cell therapies [2]. Although hard to detect, utilizing epitope spreading could help target diverse tumor tissues by enhancing effector cell migration to the TME or inducing tertiary lymphoid organs.

### 10.4 Tertiary Lymphoid Organs (TLOs)

The presence of TLOs, clusters of immune cells near tumors, has been associated with improved outcomes in vaccination and immune checkpoint blockade [212]. TLOs are identified through transcriptional profiling and multispectral imaging, although standardization and distinguishing between immature and mature TLOs still present challenges [2]. Promoting TLO formation could enhance anti‐tumor responses, while their presence might also assist in guiding patient selection for specific combination therapies.

### 10.5 Non‐Invasive Biological Signatures

***Gut microbiome as biomarker and therapeutic target***: Gut flora composition influences the response to ICIs, possibly by enhancing the release of DC‐mediated cytokines to activate systemic immune responses and by cross‐reacting with tumoral antigens [1, 212]. Commensals such as Bacteroides, Bifidobacterium, and Ruminococci are needed to induce responses to ICIs, such as anti‐CTLA4 and anti‐PD1/PD‐L1 in melanoma and liver cancer, while *Akkermansia muciniphila* is common in responders across various cancers [1, 213]. Despite the lack of a standard method for analyzing specific microbiome compositions in response to or non‐response, success in preclinical models has led to clinical studies combining gut flora with immunotherapy [215]. A phase I clinical trial manipulating bacterial composition by administering a specific probiotic alongside anti‐PD1 and anti‐CTLA4 resulted in longer progression‐free survival in patients with metastatic renal cancer [216]. Recently, multiple clinical trials involving gut microbiome and ICIs combinations are ongoing for various cancers, including melanoma, NSCLC, RCC, urothelial, prostate, and other solid tumors [214]. Gut microbiome‐based strategies hold promise for enhancing response to ICIs, ICIs efficacy, reducing treatment toxicities, and overcoming resistance in genitourinary cancers, but further well‐designed clinical trials are necessary before these approaches can be incorporated into standard care.

***Artificial Intelligence (AI)‐based biomarkers***: The most promising and clinically relevant applications of artificial intelligence (AI) in immunotherapy today lie in non‐invasive biomarkers and predictive models using standard‐of‐care imaging or liquid biopsy. Blood‐based markers, such as circulating tumor DNA (ctDNA) or circulating tumor cells are suitable for longitudinal assessments [217]. Detecting reductions in ctDNA levels early predicts better clinical outcomes in ICIs‐treated NSCLC [217, 218]. Analysis across advanced solid tumors treated with ICIs found reduction in ctDNA 6 to 16 weeks after therapy started, and was strongly associated with improved PFS and OS [220]. In NSCLC, serial ctDNA measurements during ICIs therapy demonstrated more than 50% reduction in ctDNA from baseline correlated with better survival [220, 221]. These data suggest ctDNA dynamics, when tracked longitudinally and potentially combined with machine learning models, can serve as early and minimally invasive biomarkers to ICIs. Additionally, ctDNA is used to track genetic heterogeneity and to detect MRD (minimal or molecular residual disease) after specific treatments across multiple cancer types [2, 222]. Recent studies demonstrated that the presence of metabolomic signatures in patient sera predicted response to ICIs [223]. Exploring immune gene signatures and signatures associated with immune response regulation, such as tRNA‐derived fragments, may offer potential for predicting responses to immune checkpoint inhibitors [224, 225]. However, rigorous investigation and comprehensive validation are required before their clinical utility can be established.

***Imaging***: Imaging data offer an alternative to tissue biopsies by enabling visualization of the entire tumor and metastases. Delta‐radiomics, which tracks changes in tumor features over time, could predict initial responses, non‐responses, or acquired resistance to ICIs, as well as detect tumor recurrence [227]. Machine learning models using radiomics have outperformed traditional methods, such as RECIST, in predicting ICIs responses and adverse events [2]. Recent studies demonstrate that machine learning‐based radiomic signatures derived from CT or PET‐CT imaging can predict response to ICIs across multiple cancer types. In a study of hepatocellular carcinoma patients treated with ICIs, a radiomic model based on pretreatment CT distinguished responders from non‐responders with high accuracy (86%) [228]. Similarly, for oral squamous cell carcinoma, a CT‐based radiomics model was associated with immunotherapy response [229]. In addition, a CT radiomics model could predict microsatellite‐instability state and immunotherapy outcomes in gastric cancer, where the radiomics score correlated with CD8^+^ T cell infiltration and inversely with M2 macrophages, linking imaging phenotypes to immune TME features [230]. A recent pan‐cancer radiomics signature called “CT‐TIME” was developed to non‐invasively estimate a T cell–inflamed tumor microenvironment and predict response to ICIs. In external validation, the signature achieved an AUC of 0.78 and correlated with immunohistochemistry for CD8^+^ cells [231]. These findings support radiomics as a non‐invasive, clinically usable tool for stratifying patients prior to ICIs, augmenting or potentially overcoming limitations of tissue‐based biomarkers. Radiomics also helps distinguish pseudo‐progression from true progression, thereby preventing premature treatment cessation. Thus, integrating radiomics with clinical or blood biomarkers enhances prediction accuracy [232].

Near‐term priorities for biomarker research are ctDNA dynamics for early response monitoring and minimal residual disease, and externally validated radiomic signatures that can augment tissue‐based biomarkers. Investing in prospective and harmonized biomarker pipelines, such as serial ctDNA measurement, standardized imaging, and radiomics linking with immune‐profiling, will provide pragmatic, non‐invasive tools to steer adaptive combination strategies.

## 11 Conclusion and Future Directions

While immune checkpoint inhibitors (ICIs) have advanced the field of cancer immunotherapy and their combination with other treatment modalities has achieved greater success, continued refinement of combination strategies remains essential. The integration of next‐generation immunotherapies tailored explicitly to the tumor microenvironment (TME) is expected to play a crucial role in overcoming resistance to ICIs. Additionally, immunotherapy must progress through rational combination strategies guided by biomarker insights, blending hypothesis‐driven research with exploratory analyses to understand therapeutic mechanisms and improve patient selection. Exploratory biomarker studies are vital to avoid the pitfalls of combination trials based solely on limited preclinical data, which often lead to low success rates and strain healthcare systems. Emerging approaches, including neoantigen vaccines, microbiome interventions, and targeted cell therapies, show promise and could lead to a transformative era in immunotherapy combinations when paired with advanced biomarker platforms such as circulating tumor DNA profiling, multi‐omic TME analysis, and radiomics. While current biomarkers remain exploratory, future models will need standardized, multi‐analyte algorithms that incorporate tumor genetics, immune responses, and host factors to predict and monitor treatment responses. Achieving this goal requires collaborative efforts to harmonize data from various sources, supported by robust bioinformatics pipelines. Ultimately, the integration of new technologies, biomarker validation, and adaptive trial designs will advance precision immunotherapy, enabling the development of TME‐specific combinations that overcome immune suppression and provide durable benefits for ICIs‐resistant patients. In this context, AI‐driven biomarker tools with current clinical evidence, particularly radiomics‐based imaging signatures and ctDNA dynamics enhanced by machine‐learning models, represent the most immediately actionable digital approaches. Their integration into prospective trial designs will strengthen patient stratification and real‐time treatment monitoring without overreliance on speculative technologies. Short‐term priorities should concentrate on rationally expanding combinations that already have Phase‐III support or regulatory approval, while integrating prospective pharmacodynamic/biomarker endpoints (tumor pharmacodynamics, ctDNA, radiomics) and adaptive designs to de‐risk Phase‐III programs. Simultaneously, investment in validated translational models and harmonized biomarker pipelines will be required to bring more exploratory approaches to clinical maturity.

## Author Contributions

A.P. Conceptualization: Lead, Writing – original draft: Lead, Writing – review & editing: Lead; S.R., L.M., L.H., and V.K. Writing – review & editing: Supporting; H.Y. Project administration: Lead; Y.D. Conceptualization: Lead, Funding acquisition: Lead, Project administration: Lead, Supervision: Lead, Writing – review & editing: Lead. All authors read and approved the final manuscript.

## Conflicts of Interest

The authors declare no conflict of interest.

## Data Availability

The authors have nothing to report.
